# Expression Pattern and Biological Significance of the lncRNA ST3GAL6-AS1 in Multiple Myeloma

**DOI:** 10.3390/cancers12040782

**Published:** 2020-03-25

**Authors:** Domenica Ronchetti, Katia Todoerti, Cristina Vinci, Vanessa Favasuli, Luca Agnelli, Martina Manzoni, Francesca Pelizzoni, Raffaella Chiaramonte, Natalia Platonova, Nicola Giuliani, Pierfrancesco Tassone, Nicola Amodio, Antonino Neri, Elisa Taiana

**Affiliations:** 1 Department of Oncology and Hemato-Oncology, University of Milan, 20122 Milan, Italy; domenica.ronchetti@unimi.it (D.R.); cristina.vincini@unimi.it (C.V.); vanessa.favasuli@unimi.it (V.F.); luca.agnelli@gmail.com (L.A.); martina.manzoni@unimi.it (M.M.); elisa.taiana@unimi.it (E.T.); 2 Hematology, Fondazione Cà Granda IRCCS Policlinico, 20122 Milan, Italy; katiatodoerti@gmail.com (K.T.); frapelli7@gmail.com (F.P.); 3 Department of Health Sciences, University of Milan, 20142 Milan, Italy; raffaella.chiaramonte@unimi.it (R.C.); natalia.platonova@unimi.it (N.P.); 4 Hematology and BMT Unit, Azienda Ospedaliero-Universitaria di Parma, 43125 Parma, Italy; nicola.giuliani@unipr.it; 5 Department of Experimental and Clinical Medicine, Magna Graecia University of Catanzaro, 88100 Catanzaro, Italy; tassone@unicz.it (P.T.); amodio@unicz.it (N.A.)

**Keywords:** lncRNA, ST3GAL6-AS1, multiple myeloma

## Abstract

The biological impact of long non-coding RNAs (lncRNAs) in multiple myeloma (MM) is becoming an important aspect of investigation, which may contribute to the understanding of the complex pathobiology of the disease whilst also providing novel potential therapeutic targets. Herein, we investigated the expression pattern and the biological significance of the lncRNA ST3 beta-galactoside alpha-2,3 sialyltransferase 6 antisense RNA 1 (ST3GAL6-AS1) in MM. We documented a high ST3GAL6-AS1 expression level in MM compared to normal plasma cells (PCs) or other hematological malignancies. Transcriptome analyses of MM PCs from patients included in the CoMMpass database indicated a potential involvement of ST3GAL6-AS1 in MAPK signaling and ubiquitin-mediated proteolysis pathways. ST3GAL6-AS1 silencing by LNA-gapmeR antisense oligonucleotides inhibits cell proliferation and triggers apoptosis in MM cell line. Notably, ST3GAL6-AS1 silencing in vitro displayed the down-regulation of the MAPK pathway and protein ubiquitination. These data suggest that ST3GAL6-AS1 deregulation may play a pathogenetic role in MM by affecting both proliferation pathways and circuits fundamental for PC survival. However, ST3GAL6-AS1 expression levels seem not to be significantly associated with clinical outcome and its targeting appears to exert antagonistic effects with proteasome inhibitors used in MM. These findings strongly urge the need for further studies investigating the relevance of ST3GAL6-AS1 in MM.

## 1. Introduction

Multiple myeloma (MM) is a malignant proliferation of bone marrow plasma cells (PCs) characterized by a heterogeneous clinical course, ranging from the pre-malignant condition called monoclonal gammopathy of undetermined significance (MGUS), to smoldering MM (sMM), truly overt and symptomatic MM, and extra-medullary myeloma/plasma cell leukemia (PCL). MM is also characterized by a highly heterogeneous genetic background with both structural chromosomal aberrations and specific gene mutations [1]. 

Long non-coding RNAs (lncRNAs) form a group of non-protein coding RNAs longer than 200 nucleotides, representing more than half of the mammalian non-coding transcriptome [2], the estimated number of which exceeding 120,000. LncRNAs are involved in many biological processes, such as cell proliferation, apoptosis, cellular differentiation, tumorigenesis and metastasis [2,3]. Recent findings indicate that the deregulation of lncRNAs is emerging as a new paradigm in cancer [4]. Several lncRNAs have been reported to be putatively involved in MM tumor biology and our knowledge of their function is progressively expanding [5,6,7,8,9]; however, at the moment only a few of them have been functionally investigated, including MALAT1 [10,11,12], NEAT1 [13,14], CCAT1 [15] and H19 [16,17]. MALAT1 was found upregulated during the disease progression, with the higher expression levels associated with shorter OS and PFS [11]. In details, MALAT1 interacts with EZH2 to regulate KEAP1 expression, triggering a KEAP1-dependent induction of NRF1/2, which are relevant transcriptional activators of proteasome subunit genes [10]. In addition, our group provided evidence of MALAT1 druggability using LNA-gapmeRs ASOs in vitro and in vivo in NOD-SCID mice bearing MM xenografts [10]. Hu et al. reported that MALAT1 acts as a scaffold in the formation of PARP1/LIG3 complexes that activate the alternative non-homologous end joining (A-NHEJ) DNA repair in MM cells; moreover, its inhibition can synergize both with PARP and proteasome inhibitors [12]. Concerning NEAT1, it was found upregulated in primary MM cells [14], where it plays a key role in maintenance of genomic stability and DNA repair [13]. Of translational relevance, NEAT1 targeting antagonized growth of MM cells in NOD SCID mice and synergized with conventional and novel anti-MM drugs [13]. 

Herein, we investigated the pathobiological role of ST3GAL6-AS1 (ST3 beta-galactoside alpha-2,3 sialyltransferase 6 antisense RNA 1), a lncRNA mapped at chromosome 3q11.2 antisense to *ST3GAL6* (ST3 Beta-Galactoside Alpha-2,3-Sialyltransferase 6), a gene that has been recently reported as involved in homing and in in vivo engraftment in MM [18]. A recent study in a limited cohort of MM patients, pointed out the overexpression of ST3GAL6-AS1 compared to normal controls, suggesting a putative oncogenic role [19]. However, in colorectal cancer, ST3GAL6-AS1 was reported to act as tumor suppressor through the transcriptional regulation of its neighboring gene *ST3GAL6* [20].

In the present study, we analyzed the expression pattern of ST3GAL6-AS1 in a proprietary transcriptome database including 50 MM at diagnosis and in the larger CoMMpass database [21]. Additionally, we performed in vitro studies to assess the biological effect of ST3GAL6-AS1 silencing in human MM cell lines. 

## 2. Results

### 2.1. LncRNA ST3GAL6-AS1 is Overexpressed in MM Patients 

**Figure 1:**
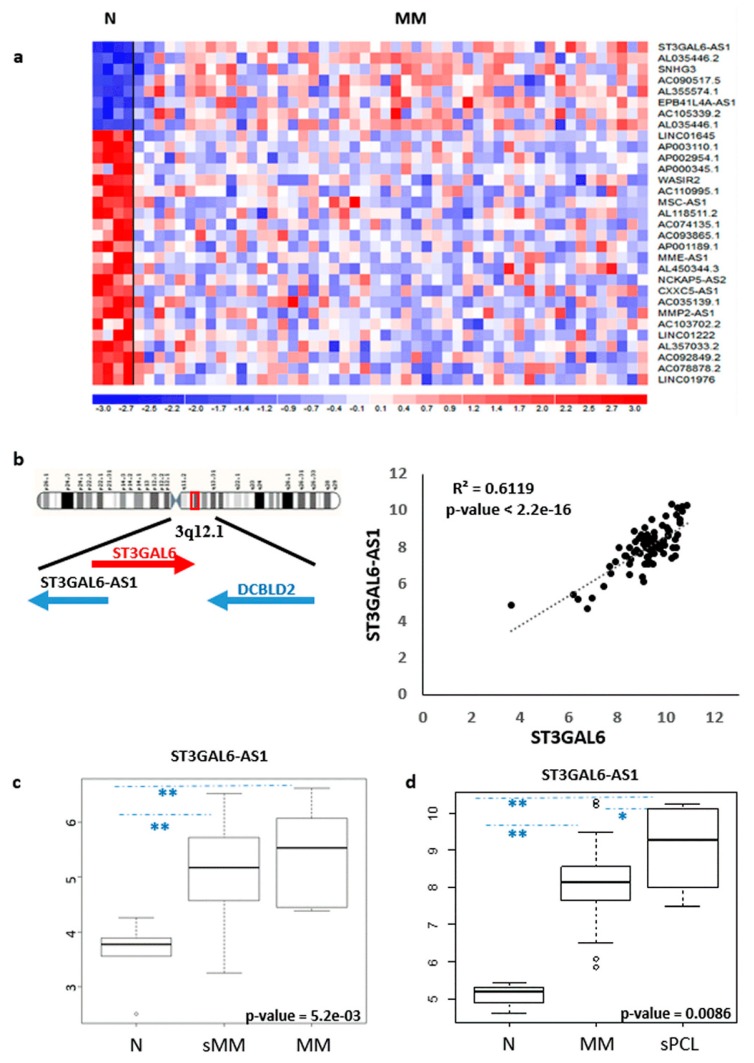
Identification of lncRNAs deregulated in MM patients. (**a**) Heatmap of the 31 differentially expressed lncRNAs in 50 MM patients as compared to four normal bone marrow PC controls (patients in columns, lncRNAs in rows). The color scale bar represents the relative lncRNA expression changes normalized by the standard deviation. (**b**) Genomic context of ST3GAL6-AS1; Pearson’s correlation analysis of *ST3GAL6* (x-axis) and ST3GAL6-AS1 (y-axis) expression levels. Correlation coefficient R and significant p-value are reported. (**c**) Boxplot of ST3GAL6-AS1 expression in five normal controls, 21 sMM and 10 MM samples (** *p* < 0.01). (**d**) Boxplot of ST3GAL6-AS1 expression in four normal controls, 50 MM, and six sPCL samples (* *p* < 0.05, ** *p* < 0.01).

With the aim of identifying lncRNAs deregulated in MM, we investigated their expression pattern in a proprietary database (#GSE116294) recently reported by us [14], including four normal controls, 50 MM patients at diagnosis and six secondary PCL (sPCL) profiled on the GeneChip^®^ Human Gene 2.0 ST array. Although limited in number, the cohort of MM was representative of the major molecular types of the disease (Table S1). To this end, we evaluated the expression of 10,500 lncRNAs detectable by the arrays, annotated on unambiguous entries in GENCODE encyclopedia (V32). We identified 31 lncRNAs showing highly significant differential expression between normal bone marrow PCs and MM patients (Figure 1a and Table S2). 

Notably, the most significantly upregulated lncRNA in our series was ST3GAL6-AS1, recently described as upregulated in MM patients in association with worse stages of the disease [19]. ST3GAL6-AS1 is located at chromosome 3q12 head-to-head with the *ST3GAL6* gene, sharing a 415 bp complementary sequence. Approximately 60Kb telomeric and in sense orientation to ST3GAL6-AS1, is located the *DCBLD2* (Discoidin, CUB and LCCL Domain Containing 2) gene encoding a receptor tyrosine kinase with aberrant expression in malignant tumors. Interestingly, Pearson’s correlation analysis revealed that ST3GAL6-AS1 expression positively correlates to that of *ST3GAL6* gene (Figure 1b), whereas *DCBLD2* showed an unrelated transcription pattern. We used a quantitative real time PCR-based (qRT-PCR) approach (see Supplementary Methods) to validate microarrays data in 38 out of the 50 MM samples, confirming the positive correlation between ST3GAL6-AS1 and *ST3GAL6* (Figure S1a,b). In addition, both ST3GAL6-AS1 and *ST3GAL6* were expressed in MM patients irrespectively of the presence of the major chromosomal aberrations, namely t(11;14), t(4;14), *MAF* gene translocations or hyperdiploid (HD) status, or the presence of *KRAS, NRAS, TP53, BRAF, FAM46C* or *DIS3* gene mutations.

The upregulation of ST3GAL6-AS1 in pathological samples was evidenced in an independent dataset of patients (GSE117847) profiled on Clariom-D array, and including five normal controls, 21 sMM and 10 MM specimens. Remarkably, not only MM but also sMM patients significantly upregulated ST3GAL6-AS1 (Figure 1c). Interestingly, we also found that the ST3GAL6-AS1 expression further increased in sPCL cases investigated by Gene 2.0 ST array (Figure 1d), suggesting that high ST3GAL6-AS1 expression level may be likely associated with the advanced stage of the disease.

**Figure 2:**
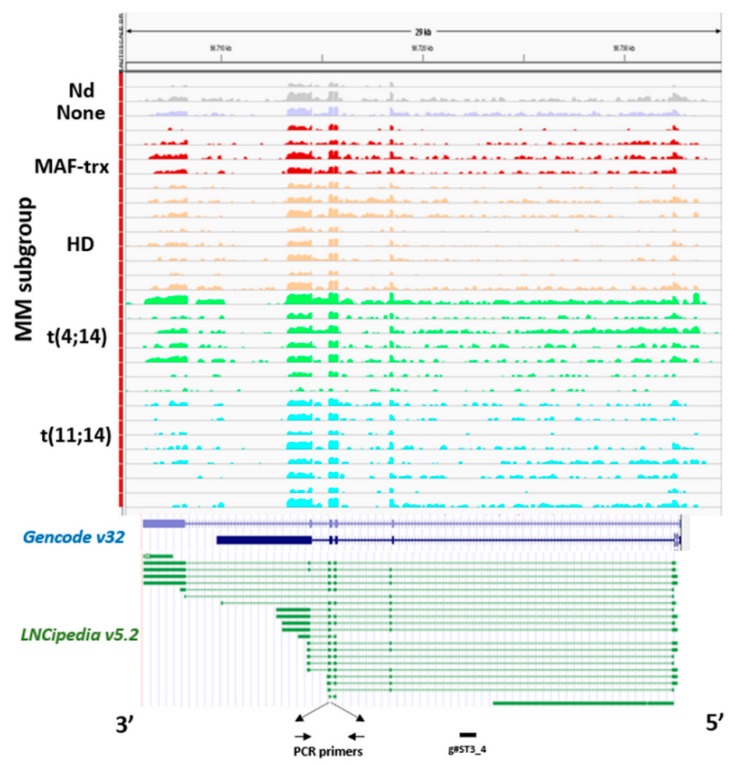
Analysis of ST3GAL6-AS1 expression. ST3GAL6-AS1 expression by RNA-sequencing. Visualization of RNA-seq data: zoomed view of the ST3GAL6-AS1 lncRNA region; the coverage bigWig files generated using bamCoverage function in deeptools [23] and the human genome annotation file (GENCODE v.32) were loaded into the Integrated Genome Viewer [24]. FANTOM CAT transcripts included in LNCipedia repository are also reported. The y-axis shows the scaled number of reads mapping to each location of the genome in the ST3GAL6-AS1 region (x-axis) schematically reported. Each lane represents a MM patient: different colors refer to the sample molecular characteristic indicated at the left. In order to compare samples, coverage values from all patients were group-scaled. Localization of primers used in RT-PCR analysis and g#ST3_4 gapmeR used for functional study is also indicated at the bottom.

ST3GAL6-AS1 expression analysis was extended to a data set obtained by RNA-sequencing (RNA-seq) of 30 out of the 50 MM cases profiled by gene expression microarrays, as previously reported [22]. In agreement with microarray data, ST3GAL6-AS1 appeared to be consistently expressed in all MM patients investigated regardless of the molecular subtypes (Figure 2). Notably, the reads alignment suggested that ST3GAL6-AS1 transcript variants are not limited to the canonical five exons, described in literature to date [19], but it could also fit with several computationally inferred variants, e.g., those from FANTOM CAT assembly included in the most recent annotations from GENCODE and LNCipedia repositories. However, although RNA-seq analysis could provide information concerning putative boundaries and junctions between contiguous exons, it does not allow defining all the potential whole-assembled variants with sufficient accuracy. Therefore, this aspect should be considered out of the scope of the present study. 

**Figure 3:**
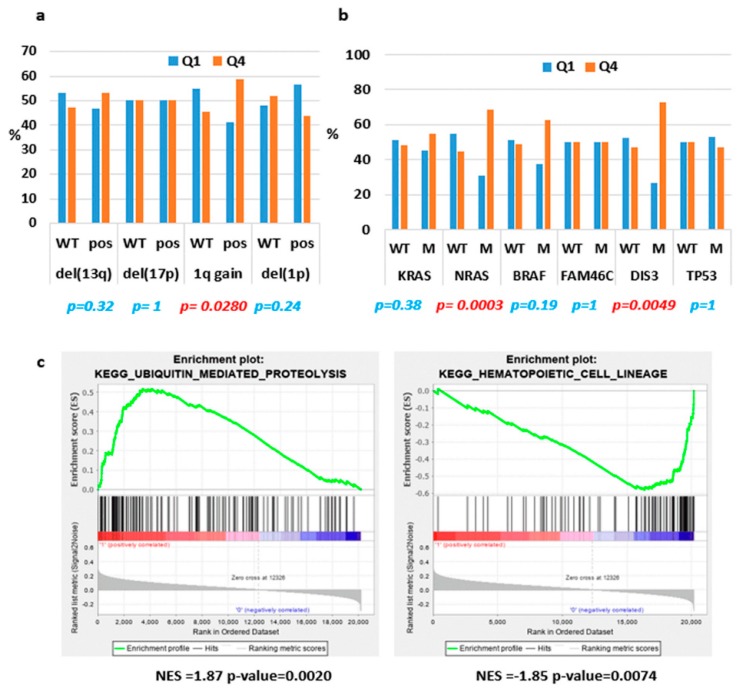
ST3GAL6-AS1 expression in CoMMpass database. (**a**,**b**) Histograms representing the occurrence of the indicated molecular features in the I and IV quartiles (Q1 and Q4) analyzed by the Fisher’s exact test; significant p-value are reported in red. (**c**) Enrichment plots of two selected gene sets detected by GSEA. The green curves show the enrichment score and reflect the degree to which each gene (black vertical lines) is represented at the bottom of the ranked gene list. NES: Normalized Enrichment Score.

Next, we extended the analyses of ST3GAL6-AS1 expression to the large MMRF CoMMpass database including 767 MM patients profiled by RNA-sequencing, whose molecular characteristics are reported in Table S3. With the aim of better defining molecular features possibly associated with ST3GAL6-AS1 expression, we grouped patients into quartiles according to ST3GAL6-AS1 expression levels (Figure S2), and compared the lowest and the highest quartile (i.e., the I and the IV quartiles including 192 patients/each group). Based on a Fisher’s exact test between these two groups, we confirmed the independence of ST3GAL6-AS1 expression from the occurrence of the main chromosomal translocations in MM. Conversely, the IV quartile group was significantly enriched in patients with chromosome 1q gain (Figure 3a and Table S4), whereas chromosome 13q, 17p, and 1p deletions were equally distributed in both quartiles. Regarding the association with *KRAS*, *NRAS*, *TP53*, *BRAF*, *FAM46C* or *DIS3* gene mutations, we found that *NRAS* and *DIS3* mutations were significantly enriched in the upper quartile group showing the highest ST3GAL6-AS1 expression (Figure 3b and Table S4). 

The same approach was used to unveil molecular pathways and putative biological effects associated with ST3GAL6-AS1 modulation. In detail, we compared the transcriptional profiles of patients belonging to the two extreme quartiles, obtaining 482 differentially expressed coding genes. Interestingly, the functional annotation analysis of this signature, aimed at identifying highly significant represented categories, revealed the enrichment in biological processes like migration and chemotaxis, positive regulation of metabolic processes, negative regulation of cell communication, positive regulation of apoptotic process, and positive regulation of MAPK cascade (Table S5). In addition, enrichment analysis of all the annotated coding-genes using Gene Sets Enrichment Analysis (GSEA) tool, evidenced that the IV quartile was significantly enriched in genes involved in ubiquitin mediated proteolysis, and negatively associated with different pathways including hematopoietic cell lineage (Table S6 and Figure 3c).

**Figure 4:**
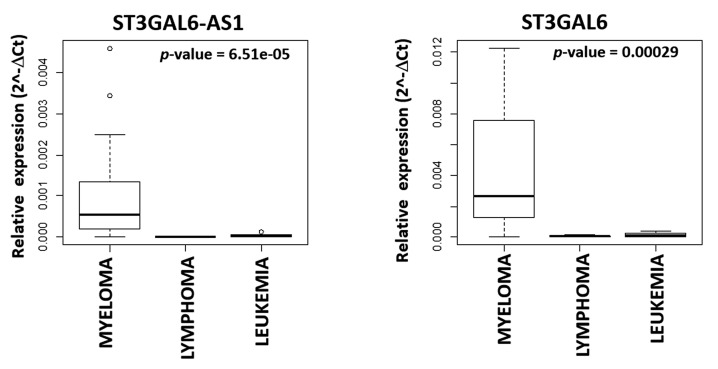
Boxplot of ST3GAL6-AS1 and *ST3GAL6* expression in 21 MM, six lymphoma, and five leukemia cell lines based on the qRT-PCR approach described in the Supplementary Methods.

Finally, we extended ST3GAL6-AS1 and *ST3GAL6* expression analysis to a representative panel of human hematological tumors, including MM (*n* = 21), lymphoma (*n* = 6) and leukemia (*n* = 5) cell lines, and found that the expression levels of both ST3GAL6-AS1 and *ST3GAL6* were significantly higher in human MM cell lines (HMCLs) as compared with other tumor types (Figure 4 and Figure S3).

Overall, the remarkably higher ST3GAL6-AS1 expression in MM plasma cells suggests that this lncRNA may play an important role in the pathobiology of the disease.

### 2.2. ST3GAL6-AS1 Expression is Unrelated to MM Prognosis

To gain insight into the relevance of ST3GAL6-AS1 expression in clinical outcome in MM, we took advantage of the CoMMpass database. Specifically, clinical and outcome data concerning Overall Survival (OS), freely accessible from MMRF Study, were analyzed in 767 MM cases. In detail, we defined two MM patient groups based on their ST3GAL6-AS1 expression level by choosing as the optimal cut-point the one that defines the most significant relation between the outcome and the ST3GAL6-AS1 continuous expression level (see Supplementary Methods). Following this approach, a Kaplan–Meier survival analysis showed that ST3GAL6-AS1 expression levels in these patients are not significantly associated with OS (Figure S4).

### 2.3. ST3GAL6-AS1 Silencing Affects Cell Cycle and Induces Apoptosis

**Figure 5:**
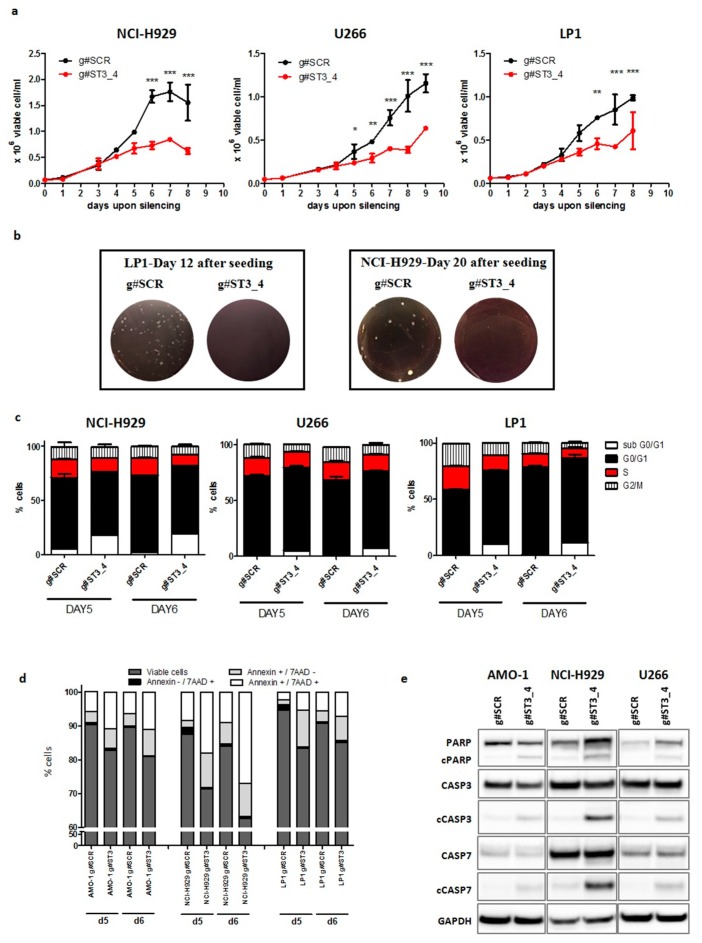
ST3GAL6-AS1 inhibition affects HMCLs cell cycle and apoptosis. (**a**) HMCLs growth inhibition upon gymnotic ST3GAL6-AS1 silencing with g#ST3_4 gapmeR (5 μM). * *p* < 0.05, ** *p* < 0.01, *** *p* < 0.001). (**b**) Colony formation assay performed on LP1 and NCI-H929 treated with g#ST3_4 gapmeR (5 μM) for 12 or 20 days, respectively; representative pictures of colonies are shown. (**c**) Histogram of cell cycle analysis by propidium iodide (PI) staining performed in NCI-H929, U266, and LP1 cells 5 and 6 days after treatment with g#ST3_4 gapmeR (5 μM). (**d**) Flow cytometry analyses of apoptosis in NCI-H929, AMO-1, and LP1 5 days after treatment with g#ST3_4 gapmeR (5 μM). (**e**) WB of cleaved caspase 3 and 7 and PARP in AMO-1, NCI-H929, and U266 6 days after delivery of g#ST3_4 gapmeR (5 μM). A representative picture of at least three experiments is shown.

To investigate the functional role of ST3GAL6-AS1 in MM, we exploited a loss of function approach by using LNA-gapmeR ASOs that triggers RNAse-H-dependent degradation of lncRNAs. Specifically, we designed “in-house” four sequences able to recognize the majority of the ST3GAL6-AS1 transcripts and tested them in NCI-H929, LP1, and U266 HMCLs by using electroporation (Figure S5a). Among them, the g#ST3_4 gapmeR showed the strongest silencing efficiency (nearly 50%) after 72 h. To achieve a more pronounced and prolonged knockdown (KD), we improved our in vitro experimental condition by optimizing the gymnotic delivery of the selected g#ST3_4 gapmeR. A downregulation efficiency reaching 97% was obtained in all tested samples (Supplementary Figure S5b), suggesting this strategy as valuable tool for subsequent investigations. In particular, we found that ST3GAL6-AS1 KD significantly reduced cell growth in NCI-H929, LP1 and U266 cell lines (Figure 5a). Accordingly, g#ST3_4 treatment dramatically suppressed the clonogenic potential of MM cells (Figure 5b). To further support the results on cell growth, we performed cell cycle analysis on ST3GAL6-AS1-depleted MM cells. As shown in Figure 5c, after five and six days of ST3GAL6-AS1 KD, an appreciable modulation of the cell cycle phases distribution was observed, with a decrease in S-G2/M phases, and an increased percentage of sub-G0/G1 phase events in NCI-H929, LP1 and U266 silenced cells (Figures S6–S8), this suggesting a pro-apoptotic effect of the LNA-gapmeR. The effect of ST3GAL6-AS1 on apoptosis was next analyzed by flow cytometry after five and six days of ST3GAL6-AS1 KD, revealing that apoptotic rate of NCI-H929, AMO-1, and LP1 silenced cells was significantly increased when compared to relative negative control (Figure 5d). Accordingly, a significant caspase 3 and 7 cleavage was observed by WB, also showing PARP cleavage upon ST3GAL6-AS1 silencing (Figure 5e and Figure S10). 

### 2.4. Identification of ST3GAL6-AS1 Targets in MM Cells

**Figure 6:**
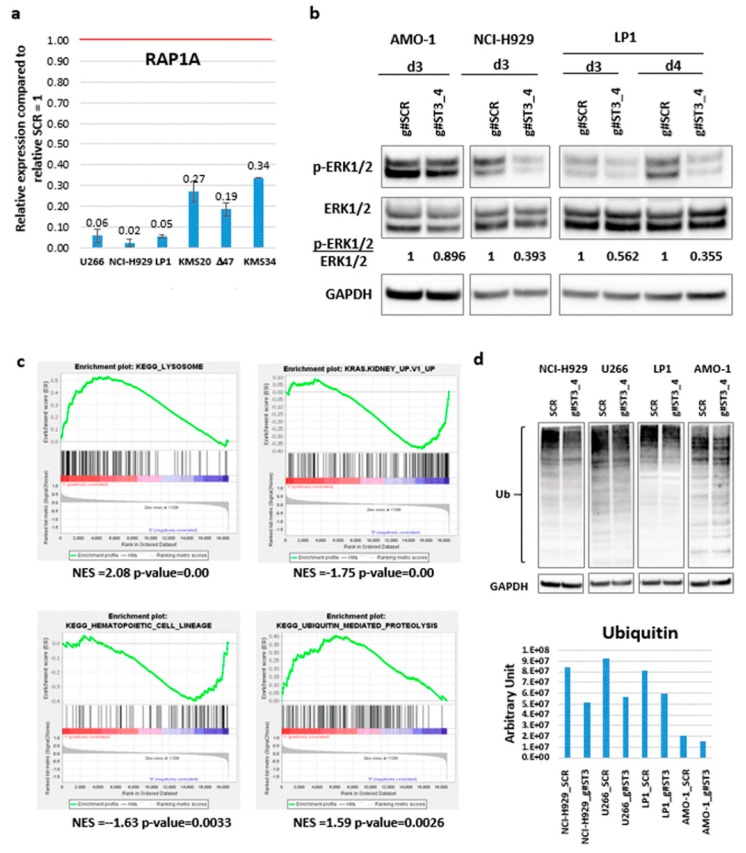
g#ST3_4 LNA-gapmeR effect on HMCLs. (**a**) *RAP1A* expression validation by RT-PCR. Mean and standard deviation on three replicates are reported. (**b**) WB of p-ERK1/2 and total ERK1/2 after delivery of g#ST3_4 gapmeR (5 μM) at the indicated time points. HMCLs were cultured in FBS 1%. (**c**) Enrichment plots of four selected gene sets detected by GSEA. The green curves show the enrichment score and reflect the degree to which each gene (black vertical lines) is represented at the bottom of the ranked gene list. (**d**) WB of ubiquitin after treating the indicated HMCLs with g#ST3_4 gapmeR (5 μM, 3 days); densitometric evaluation is reported in the histogram below.

In order to identify ST3GAL6-AS1 downstream-related pathway in MM, we carried out gene expression profiling of NCI-H929 and LP1 cells after four days of gymnotic delivery of the g#ST3_4 gapmeR. Notably, upon ST3GAL6-AS1 silencing, expression levels of both ST3GAL6 and DCBLD2 remain unchanged. By comparing the transcriptional profiles of both ST3GAL6-AS1-silenced HMCLs to controls, we obtained 131 differentially expressed genes, 41 of which downregulated in ST3GAL6-AS1 KD cell lines (Table S7). Interestingly, the most significantly downregulated gene upon ST3GAL6-AS1 KD was *RAP1A*, encoding a member of the Ras family of small GTPases that regulates signaling pathways affecting cell proliferation and adhesion, including MAP kinase activity. Based on these results, first we validated *RAP1A* gene downregulation by qRT-PCR in NCI-H929 and LP1 and in four other HMCLs treated with g#ST3_4 gapmeR (Figure 6a). Next, we investigated the modulation of the MAP signaling pathways and found a reduction in phosphorylated MAP kinase signal following ST3GAL6-AS1 KD (Figure 6b and Figure S10).

**Figure 7:**
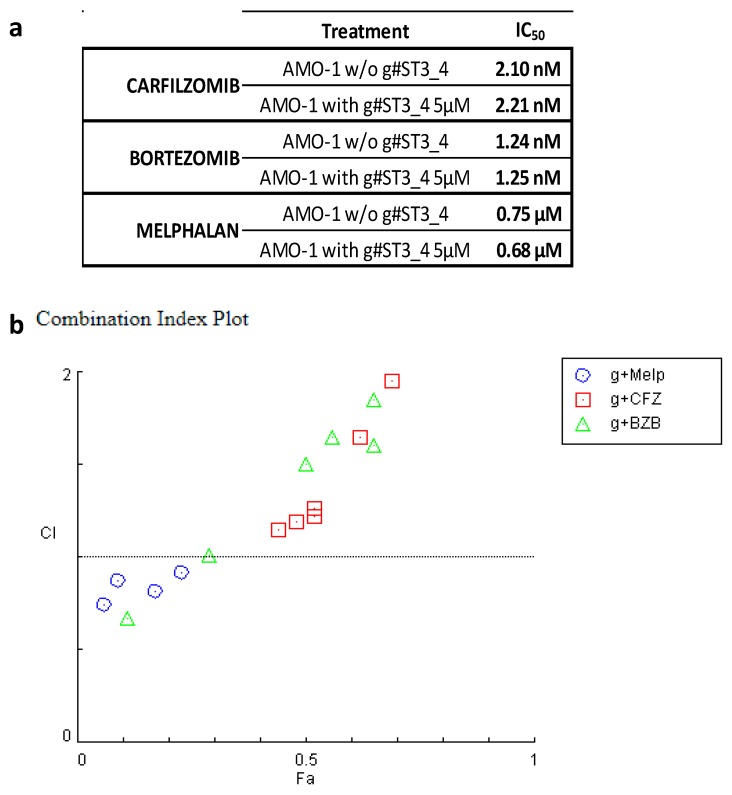
(**a**) Sensitivity of AMO-1 to BZB, CFZ, and Melph; IC_50_ was evaluated 6 days after treatment. (**b**) Chou-Talalay plots (Fa-CI) showing combination indexes (CI) resulting from combined treatments of AMO-1 with g#ST3_4 gapmeR and CFZ, Melph, or BZB (6-day time point).

Interestingly, the functional annotation analysis of the 131-gene signature revealed in particular the enrichment of two processes also resulting from the analyses of the CoMMpass database, namely the negative regulation of cell communication, and the programmed cell death (Table S8). In addition, GSEA analysis of all the annotated protein coding-genes revealed that ST3GAL6-AS1 KD signature was significantly enriched in genes involved in lysosome, and negatively correlated with genes up-regulated in epithelial kidney, lung and breast cancer cell lines over-expressing an oncogenic form of KRAS. Notably, the ST3GAL6-AS1 KD signature was significantly enriched in genes negatively associated with hematopoietic cell lineage and involved in ubiquitin-mediated proteolysis, as described above for the CoMMpass database (Figure 6c and Table S9). The relationship between ST3GAL6-AS1 expression and the ubiquitin pathway was further validated in four HMCLs, showing that ST3GAL6-AS1 KD leads to an extensive reduction in ubiquitinated protein amount (Figure 6d and Figure S10). Prompted by this observation, we wondered whether suppressing ST3AGL6-AS1 may have effects on the activity of drugs currently used in MM treatment. Hence, we evaluated the possible modulation of sensitivity of AMO-1 ST3GAL6-AS1 KD cells to bortezomib (BZB), carfilzomib (CFZ), and melphalan (Melph). Thus, we treated cells with different concentrations of these drugs in the presence or absence of ST3GAL6-AS1 gapmeR and assessed the number of viable cells in each sample for up to six days. As suggested by the IC_50_ values shown in Figure 7a, g#ST3_4 LNA-gapmeR treatment does not increase drug-sensitivity in the AMO-1 cell line; but, as observed for BZB and CFZ, it resulted in antagonistic effects (Figure 7b and Figure S9).

## 3. Discussion

Despite accumulating evidence on specific transcriptional patterns and functional roles of lncRNAs in MM [5,6,8,9,22,25], their impact on the pathobiology of the disease remains to be fully elucidated.

In this study, we have focused on ST3GAL6-AS1, a lncRNA that we found as the most significantly upregulated in MM as compared to normal bone marrow PCs. Interestingly, ST3GAL6-AS1 appears to be also significantly upregulated in sMM and further increases its expression level in sPCLs, which represent the most advanced and aggressive forms of the disease, thus suggesting a potential association with disease progression (Figure 1c,d). In addition, among different hematological tumors, ST3GAL6-AS1 deregulation appears specifically related to PC disorders (Figure 4 and Figure S3). However, based on our analyses on the CoMMpass database, ST3GAL6-AS1 expression levels do not appear to be significantly associated with clinical outcome.

Our data showed that ST3GAL6-AS1 expression was positively correlated with that of its neighboring gene *ST3GAL6*, coding for a sialyltransferase enzyme involved in homing and in in vivo engraftment in MM [18]. Such a correlation has also been recently described in colorectal cancer (CRC), where ST3GAL6-AS1 was demonstrated to regulate *ST3GAL6* transcription by recruiting histone methyltransferase MLL1 to its promoter region. Moreover, ST3GAL6-AS1 and *ST3GAL6* are down-modulated in CRC, suggesting that the ST3GAL6-AS1/*ST3GAL6* axis may play a tumor-suppressor role [20]. Overall, these data suggest that ST3GAL6-AS1 may have different biological roles in specific cellular context. 

Focusing on MM, we found that ST3GAL6-AS1 expression level was independent from the presence of the main genetic aberrations defining MM prognostic groups, apart from chromosome 1q gain, and *NRAS* or *DIS3* gene mutations as shown in the large cohort of MM patients included in the CoMMpass database, (Figure 3a,b). Particularly intriguing is the finding concerning the association between high ST3GAL6-AS1 expression level (IV quartile) and presence of *NRAS* mutations, which has been supported by further evidences. First, the functional annotation analysis of the genes significantly differentiating the upper quartile in comparison to the lowest one in the CoMMpass database showed that MM patients with higher ST3GAL6-AS1 expression levels have a gene signature related to the positive regulation of MAPK cascade (Table S5). Additionally, GSEA analysis in HMCLs upon ST3GAL6-AS1 KD evidenced that low ST3GAL6-AS1 expression levels are associated with gene set downregulation in cancer cell lines carrying an oncogenic form of KRAS (Table S9). Furthermore, ST3GAL6-AS1 silenced cells displayed reduced cellular growth and clonogenic potential, together with an increased in sub G0/G1 phase of the cell cycle associated with apoptosis induction (Figure 5). Finally, the gene expression signature of ST3GAL6-AS1 KD HMCLs showed a significant downregulation of *RAP1A*, a member of the Ras gene family [26]. Accordingly, we detected a reduction in the activated MAPK fraction by WB in ST3GAL6-AS1 KD cells (Figure 6). Overall, ST3GAL6-AS1 appeared to be involved in the MAPK signaling pathways leading to cellular proliferation, through mechanisms that warrant further studies. 

Interestingly, our data indicate that ST3GAL6-AS1 was not only implicated in proliferation pathways. In fact, GSEA analyses in patients belonging to the extreme quartiles in the CoMMpass database revealed that ST3GAL6-AS1 expression signature included genes involved in ubiquitin-mediated proteolysis, a finding that was confirmed by GSEA analysis in HMCLs upon ST3GAL6-AS1 KD. Furthermore, ST3GAL6-AS1 KD cells displayed a substantial decrease in the ubiquitinated protein amount by WB analysis. The ubiquitin pathway is crucial in MM PCs because they have the imperative need for an intact protein synthesis-folding-degradation axis given their abundant synthesis and secretion of a monoclonal immunoglobulin, or free light chain [27]. From this perspective, ST3GAL6-AS1 expression in MM may play a role in the survival of PCs. In line with this, it is worth noting that patients from the CoMMpass database with the highest ST3GAL6-AS1 expression were significantly associated with 1q gain. In our previous studies, we reported that the gene expression signature of MM patients with 1q gain was involved in processes of intracellular protein transport, and in the complex network leading to ER stress-induced responses [28]. In this context, both alterations may cooperate for the maintenance of protein homeostasis in malignant PCs. Of note, we found under our in vitro experimental condition that ST3GAL6-AS1 silencing may exert an antagonistic effect if combined with proteasome inhibitors, such as BZB and CFZ. Although these findings need to be further investigated, one possible explanation might be that a consistent reduction in protein ubiquitination (as observed in ST3GAL6-AS1 silencing) could lead to a lesser amount of substrate being addressed to the proteasome machinery, the inhibition of which may ultimately result in being less effective. 

## 4. Materials and Methods 

Full details of cell cultures, Anti-Sense Oligonucleotide (ASO) LNA gapmeRs and cell electroporation, quantitative real-time PCR, Microarray gene expression profiling, Multi-omics data in CoMMpass study, Statistical analysis, are provided in the Supplementary Methods. 

### 4.1. Samples

We investigated ST3GAL6-AS1 in a proprietary dataset that includes 4 normal bone marrow PCs purified from normal individuals (purchased from Voden, Medical Instruments IT) and MM tumors including 50 MM patients and 6 secondary PCLs (sPCL) (#GSE116294) (Table S1). All the samples, upon written informed consent, were characterized for the ploidy status, the presence of 17p13 or 13q14 deletions, 1q gain and the most frequent IGH chromosomal translocations t(11;14), t(4;14), t(14;16) and t(14;20), and the mutations pattern of *BRAF*, *NRAS*, *KRAS*, *P53*, FAM46C and *DIS3* genes as previously described [29,30,31,32]. Written informed consent was obtained from all patients in accordance with the declaration of Helsinki. The study was approved by the Ethical Committee of the University of Milan (N°24/15, May 06 2015).

### 4.2. Gymnosis

Cells were seeded at low plating density (5 × 10^4^/mL) to reach confluence on the final day of the experiments (day 6). Cells were treated with the naked gapmeRs g#ST3_4 and the scrambled g#SCR, at the same time of the seeding at a final concentration of 2.5 and 5 µM. 

### 4.3. Colony Forming Assay

For colony-forming assay, g#SCR and g#ST3_4 treated cells were analyzed as previously described [13].

### 4.4. Cell Cycle Analysis and Apoptosis

Cell cycle distribution and apoptosis of treated HMCLs were investigated as previously described [13]. 

### 4.5. Western Blot and Antibodies 

Cells were homogenized in lysis buffer M-PER^®^ Mammalian Protein Extraction Reagent (Thermo Scientific, Waltham, MA, USA) and Halt Protease and Phosphatase inhibitor cocktail, EDTA-free, 100X, (Thermo Scientific, Waltham, MA, USA). Western blot was performed as reported in Supplementary Methods.

### 4.6. Synergism Quantification

Detailed information can be found in Supplementary Methods. 

### 4.7. Gene Expression Profiling

All MM and PCL patients along with the 4 BM PC normal controls were profiled on GeneChip^®^ Human Gene 2.0 ST arrays (Affymetrix Inc., Santa Clara, CA, USA). The expression levels of 10,500 unique lncRNAs were obtained (see Supplementary Methods). The list of differentially expressed genes or lncRNAs between high and low ST3GAL6-AS1 expressing samples was gathered by Significant Analysis of Microarrays v5.00, as previously described [25]. Microarray data were globally analyzed by Gene Set Enrichment Analysis (GSEA, software v.4.0.2, Broad Institute, San Diego, CA, USA) (see Supplementary Methods) [33]. Gene sets were considered significant with nominal *p*-value < 0.05. All the data have been deposited in the NCBI Gene Expression Omnibus database [34] and are accessible under accession #GSE116294. 

### 4.8. RNA Sequencing (RNA-seq)

The RNA sequencing data have been previously generated and were publicly available at GEO repository under accession #GSE109342. Transcript abundance was estimated as previously described [22].

## 5. Conclusions

Our results indicate that ST3GAL6-AS1 deregulation may play a pathogenetic role in MM by affecting proliferation pathways and, at the same time, circuits fundamental for PC survival, such as MAPK signaling and protein ubiquitination. However, our present data seem to indicate that ST3GAL6-AS1 expression levels are not significantly associated with clinical outcome and that its targeting may have antagonistic effects with proteasome inhibitors currently used in MM. Taken together, these findings strongly suggest to investigate further the biological role and activity of ST3GAL6-AS1 in MM. 

## Supplementary Materials

The following are available online at https://www.mdpi.com/2072-6694/12/4/782/s1, Supplementary Methods. Figure S1: (a) Pearson’s correlation on ST3GAL6-AS1 (x-axis) and *ST3GAL6* (y-axis) gene expression levels in 38 MM cases analyzed by qRT-PCR. (b) Pearson’s correlation on ST3GAL6-AS1 transcript expression levels measured by qRT-PCR (x-axis) and microarray (y-axis). Correlation coefficients R and significant *p*-values are reported in both plots. Figure S2: Histogram of ST3GAL6-AS1 expression level in 767 MM patients analyzed by RNA-sequencing in CoMMpass database. Samples are ordered according to increasing ST3GAL6-AS1 expression level and grouped into quartiles; 192 patients belonging to the two extreme quartiles are boxed in red. Figure S3: ST3GAL6-AS1 and *ST3GAL6* expression in hematological tumors including 21 HMCLs, 6 lymphomas (1 Diffuse Large B-Cell Lymphoma, 3 Mantle Cell Lymphomas, 2 B-Cell Lymphomas), and 5 leukemias (1 Chronic Lymphatic Leukemia, 1 Chronic Myeloid Leukemia, 2 T-cell Acute Lymphoblastic Leukaemia, 1 B-Cell Acute Lymphoblastic Leukemia). Figure S4: ST3GAL6-AS1 expression is unrelated to MM prognosis (OS) in CoMMpass database. Kaplan—Meier estimated curves of ST3GAL6-AS1 expression. MM are stratified into low or high-expressing groups. Confidence intervals are plotted. Log-rank p-value and a table risk with the absolute number of subjects at risk by time in each group are reported. Figure S5: (a) Scheme of the 4 GapmeRs localization (Exiqon); (b) qRT-PCR analysis of ST3GAL6-AS1 silencing efficiency after 5 day from the gymnotic delivery of selected g#ST3_4 in the indicated HMCLs. Figure S6: Cell cycle analysis by PI staining was performed in LP1 cells after treatment with g#ST3_4 gapmeR (5 μM). Figure S7: Cell cycle analysis by PI staining was performed in U266 cells after treatment with g#ST3_4 gapmeR (5 μM). Figure S8: Cell cycle analysis by PI staining was performed in NCI-H929 cells after treatment with g#ST3_4 gapmeR (5 μM). Figure S9: ST3GAL6-AS1 KD antagonizes with CFZ or BZB, but not with Melph. (a) Raw-data of combination indexes (CI) resulting from combinatorial treatments of AMO-1 with g#ST3_4 and CFZ or Melph or BZB (6-day time point): fraction of viable AMO-1 cells after six days of treatment with g#ST3_4 (2,5 μM and 5 μM) and indicated concentrations of CFZ, Melph or BZB. (b) Table showing combination indexes (CI) resulting from combinatorial treatments of AMO-1 with g#ST3_4 and CFZ, Melph, or BZB (6-day time point). Figure S10: Uncropped western blots membrane acquisition showing all the bands with molecular weight markers for the indicated Figure and panel. Densitometry readings and intensity ratio of each band are provided for ubiquitin and ERK1/2 signal acquisition, respectively. Table S1: molecular characteristics of 50 MM patients. Table S2: List of the 31 differentially expressed lncRNAs identified by SAM (Significant Analysis of Microarray) analyses comparing plasma cells from MM patients with those from normal donors, ordered based on (d)-score. Table S3: Number and relative frequency of main IgH translocations inferred from spike expression of the target genes in all 767 BM-1 MM cases analyzed by RNA sequencing; major copy number alterations (CNAs) and non-synonymous somatic mutations in BM-1 MM cases for which CNAs and mutation counts data were also available by NGS-based FISH and Whole Exome sequencing (WES) analyses, respectively. Table S4: Number and relative frequency of main IgH translocations, CNAs and non-synonymous somatic mutations in BM-1 MM cases of ST3GAL6-AS1 quartile I and IV, respectively. Table S5: List of the significantly enriched Annotation Clusters for the 482 differentially expressed protein-coding genes (Enrichment Score >2) by DAVID 6.8 Bioinformatics tool. Table S6: KEGG gene sets obtained using Gene Sets Enrichment Analysis showing significant modulation between ST3GAL6-AS1 IV Quartile and ST3GAL6-AS1 I Quartile in CoMMpass database. (NES >1.5 or <−1.5; *p*-value < 0.05, NES: Normalized Enrichment Score). Table S7: Down-modulated (blue) and up-regulated (red) genes resulting from SAM analyses comparing ST3GAL6-AS1 silenced and scramble NCI-H929 and LP1 cell lines, ordered based on (d)-score. Genes at *q*-value = 0 were marked in bold. Table S8: List of the significantly enriched Annotation Cluster for the 131 differentially expressed coding genes (Enrichment Score > 1.3) by DAVID 6.8 Bioinformatics tool. Table S9: C6 and KEGG gene sets obtained using Gene Sets Enrichment Analysis showing significant modulation between ST3GAL6-AS1 KD HMCLs and control. (NES >1.5 or < −1.5; *p*-value < 0.05, NES: Normalized Enrichment Score).
