# Supplementary material for: Expression Pattern and Biological Significance of the lncRNA ST3GAL6-AS1 in Multiple Myeloma
Source: Cancers (Basel). 2020 Mar 25;12(4):782. doi: 10.3390/cancers12040782 (PMC7225964; doi:10.3390/cancers12040782)
Supplement: Supplementary file 1 [file cancers-12-00782-s001.pdf]

Supplementary Materials: Expression Pattern and Biological Significance of the lncRNA ST3GAL6-AS1 in Multiple Myeloma

Domenica Ronchetti, Katia Todoerti, Cristina Vinci, Vanessa Favasuli, Luca Agnelli, Martina Manzoni, Francesca Pelizzoni, Raffaella Chiaramonte, Natalia Platonova, Nicola Giuliani, Pierfrancesco Tassone, Nicola Amodio, Antonino Neri and Elisa Taiana

Supplementary Methods

MM Cell Lines

Human cell lines NCI-H929, U266, AMO-1 and LP1 were purchased from DSMZ, which certified authentication performed by short tandem repeat DNA typing. All these cell lines were immediately frozen and used from the original stock within 6 months. Human myeloma cell lines were cultured in RPMI-1640 medium (Gibco®, Life Technologies, Carlsbad, CA, USA) supplemented with 10% fetal bovine serum, 100 U/mL penicillin, and 100 mg/mL streptomycin (Gibco®, Life Technologies, Carlsbad, CA, USA) at 37 °C in 5% CO_2 atmosphere, and tested for mycoplasma contamination.

Design of LNA Oligonucleotides and in Vitro Transfection of HMCLs

LNA oligonucleotides were provided by Exiqon (Vedbaek, Denmark). Custom Long Non-Coding LNA gapmeRs were custom-designed and purified by HPLC followed by Na^+-salt exchange and lyophilization.

Name	Design ID	Sequence 5′-3′	Mw, calc (Da)
g#ST3_1	613937-1	AGCATAAAGAGTCTGT	5282.2
g#ST3_4	676401-1	TTTGCGTGGCTTGGAA	5324.2
g#ST3_5	676401-4	TCTGAACCCATTGAGT	5251.2
g#ST3_6	676401-7	TAATTTGCTTAGGTCA	5267.2
g#SCR		GCTCCCTTCAATCCAA	5184.2

HMCLs were transfected by Neon Transfection System (Invitrogen, Carlsbad, CA, USA), with the following electroporation conditions: 1100 V, 30 ms, 2 pulse. LNA gapmeRs were used at 100nM. The transfection efficiency was evaluated by comparative qRT-PCR.

Reverse Transcription and Quantitative PCR

Total RNA was extracted using TRIzol® Reagent (Invitrogen, Carlsbad, CA, USA) according to manufacturer's instructions. The purity and concentration of total RNA was determined by the NanoDrop 1000 spectrophotometer (Thermo Fisher Scientific, Waltham, MA, USA). The ratios of absorption (260 nm/280 nm) of all samples were between 1.8 and 2.0. cDNA was synthesized from 500 ng of total RNA with random primers using the High Capacity cDNA Reverse Transcriptase Kit (Invitrogen, Life Technologies, Carlsbad, CA, USA) according to the manufacturer's instructions. To evaluate the expression levels of listed genes, RT-PCR was performed using SYBR green PCR Master Mix (Applied Biosystems, Foster City, CA, USA) after optimization of the primer conditions. 10 ng of reverse-transcribed RNAs were mixed with 300 nM of specific forward and reverse primers in a final volume of 10 μL. RT-PCR was performed on an Applied Biosystems StepOnePlus Real-Time PCR system for 40 cycles. Data were analyzed using the $2^{-\Delta\Delta Ct}$ method to measure the relative changes in each gene's expression compared with GAPDH expression. To determine RNA levels by qPCR, the following primers were used:

Primer Name	Sequence (5′–3′)
ST3GAL6-AS1_4 FW	5′-GCAGCACAGAATCCTGACAA-3′
ST3GAL6-AS1_4 RW	5′-GCCAGCATTTTGGTAAGAGC-3′

RAP1A FW	5′-CGTGAGTACAAGCTAGTGGTCC-3′
RAP1A RW	5′-CCAGGATTTCGAGCATACACTG-3′
GAPDH FW	5′-ACAGTCAGCCGCATCTTCTT-3′
GAPDH RW	5′-AATGAAGGGGTCATTGATGG-3′

Gene Expression Profiling

GEP were obtained after 96 h of gymnotic delivery of g#ST3_4 (5 μM) or g#SCR in LP1 and NCI-H929 in three parallel experiments. RNA was extracted by RNeasy Mini kit (Qiagen, Hilden, Germany). The integrity, quality and quantity of RNA were assessed by Agilent Bioanalyzer 2100 (Agilent Technologies, Santa Clara, CA, USA) and NanoDrop 1000 Spectrophotometer (Thermo Scientific, Wilmington, DE, USA). 250 ng of RNA were used as starting material for preparing the hybridization target according to manufacturer's protocol (GeneChip® WT PLUS Reagent Kit, Thermo Scientific, Wilmington, DE, USA). Transcriptional data were generated on GeneChip® Human Gene 2.0 ST Array (Thermo Scientific, Wilmington, DE, USA) exactly as previously described [1]. Normalized expression values were obtained using Robust Multi Array Average (RMA) procedure and annotations based on GENCODE v32, Ensembl v98 (Chip Definition File available at http://brainarray.mbni.med.umich.edu/Brainarray/Database/CustomCDF/24.0.0/gencodeg.asp). CDF file was customized in order to withdraw probes that map to regions where ambiguous detection due to transcript overlap might occur.

Proteomic Assays

Whole cell lysates (40 μg per cell line) from silenced cell lines were separated using Bolt™ 4–12% Bis-Tris Plus Acrylamide Gels (Invitrogen, Carlsbad, CA, USA), electro-transferred onto nitrocellulose membranes (Bio-Rad, Hercules, CA, USA), and immunoblotted with the caspase 3 (casp3) (Cell Signaling, Danvers, MA, USA), PARP (Cell Signaling, Danvers, MA, USA), ERK and p-ERK (Sigma Aldrich, St. Louis, MO, USA), GAPDH (Santa Cruz, Santa Cruz, CA, USA) and caspase 7 (casp7) (Cell Signaling, Danvers, MA, USA) primary antibodies. Membranes were washed three times in PBST solution and then incubated with a secondary antibody conjugated with horseradish peroxidase (HRP) in BSA 2%—PBST for 2 hours at RT. Chemiluminescence was developed using Clarity ECL Western Blot Substrate Kit (BIO-RAD, Hercules, CA, USA) and signal intensity was detected by the use of ChemiDoc MP System (Bio-Rad, Hercules, CA, USA).

Synergism Quantification and Drugs

Clinical-grade bortezomib, carfilzomib, and Melphalan were purchased from Selleckchem (Munich, Germany) and solved in DMSO as recommended at a concentration of 5mM. Drug combination studies and their synergy quantification followed the Chou-Talalay method. First, dose-effect curves were determined in AMO-1 after six days of treatment for each drug, including carfilzomib, melphalan, or bortezomib. Then, different concentrations of each anti-MM agent were combined to g#ST3_4 gapmeR ranging 2,5–5μM. Combination indexes (CI) were calculated by CalcuSyn (BIOSOFT, Cambridge, UK).

Multi-Omics Data in CoMMpass Study

Multi-omics data about baseline newly-diagnosed multiple myeloma (MM) patients (BM_1) enrolled in Multiple Myeloma Research Foundation (MMRF) CoMMpass Study were publicly available (https://research.themmrf.org/) and extracted from the Interim Analysis 12a (MMRF_CoMMpass_IA12a).

Transcript per Million (TPM) reads values, obtained by Salmon gene expression quantification (MMRF_CoMMpass_IA12a_E74GTF_Salmon_V7.2_Filtered_Gene_TPM), were achieved in 767 BM_1 MM patients profiled by RNA sequencing (RNA-seq). The occurrence of the main canonical IgH translocations were inferred from RNA-seq spike expression estimates of known target genes (MMRF_CoMMpass_IA12a_RNAseq_Canonical_Ig_Translocations); copy number alterations

(CNAs) data were obtained by means of Next generation Sequencing (NGS)-based fluorescence *in situ* hybridization (FISH) analyses (MMRF_CoMMpass_IA12a_CNA_Exome_FISH_CN_All_Specimens) [2]. Non-synonymous (NS) somatic mutation counts were derived from whole exome sequencing (WES) data (MMRF_CoMMpass_IA12a_All_Canonical_NS_Variants_ENSG_Mutation_Counts).

Statistical Analysis

Kruskal Wallis test was applied for ST3GAL6-AS1 differential expression analysis and dunn's test for multiple comparisons between PC dyscrasia groups.

Fisher's exact test was applied to verify the association of the major genomic aberrations with ST3GAL6-AS1 expression levels. The two-tailed p-value < 0.05 was considered statistically significant.

Kaplan-Meier method was applied on overall survival (OS) data in 767 BM_1 MM patients of CoMMpass dataset (CoMMpass_IA12_FlatFiles). To define high- and low-expressing groups, an optimal cut-point corresponding to the most significant relation with outcome was determined on ST3GAL6-AS1 continuous expression level, by means of the maximally selected rank statistics (maxstat), as implemented in R Bioconductor (v 3.5.1) survminer package.

Long-rank test p-value was calculated between ST3GAL6-AS1 high and low groups and patients were stratified in each group according to their risk by time.

Differential Expression and Functional Enrichment Analyses

RNA-seq gene expression data were annotated using MMRF annotation file (Homo_sapiens.GRCh37.74.gtf) and only 20.276 protein coding genes were considered for further analyses. According to ST3GAL6-AS1 expression level, all 767 BM_1 MM cases were stratified in quartiles and the extreme ones (192 MM samples in 4th versus 192 MM in 1st quartile) were compared. A pre-filtering step was applied, thus considering for differential expression analysis only genes with at least 1 TPM reads value in at least 5% of the samples (5019/20276, 25% protein coding genes filtered out). In order to fit a linear model to each gene, a least squares approach was applied on log2-transformed expression data, by using limma package [3] in R Bioconductor (v 3.5.1). Differential expressed protein coding genes were selected at 10% FDR adjusted p-value and 1.5 fold change cut-off values.

Gene set enrichment analysis (GSEA) analysis [4] was performed on total protein coding genes, using KEGG or C6 oncogenic signatures collections (v7.0), by setting 5-500 gene set dimensions and performing 1000 permutations on samples (CoMMpass database) or gene sets (HMCLs), respectively.

Functional annotation clustering of Biological Process (BP), Molecular Function (MF) and Cellular Component (CC) Gene ontology (GO) terms was performed on differentially expressed gene lists, using The Database for Annotation, Visualization and Integrated Discovery (DAVID) 6.8 Bioinformatics tool [5,6]. Significant Annotation Clusters were selected on the base of Enrichment Score values (ES > 2, p-value < 0.01 or ES > 1.3, p-value < 0.05) and the most significant and representative GO-terms were reported for each Annotation Cluster.

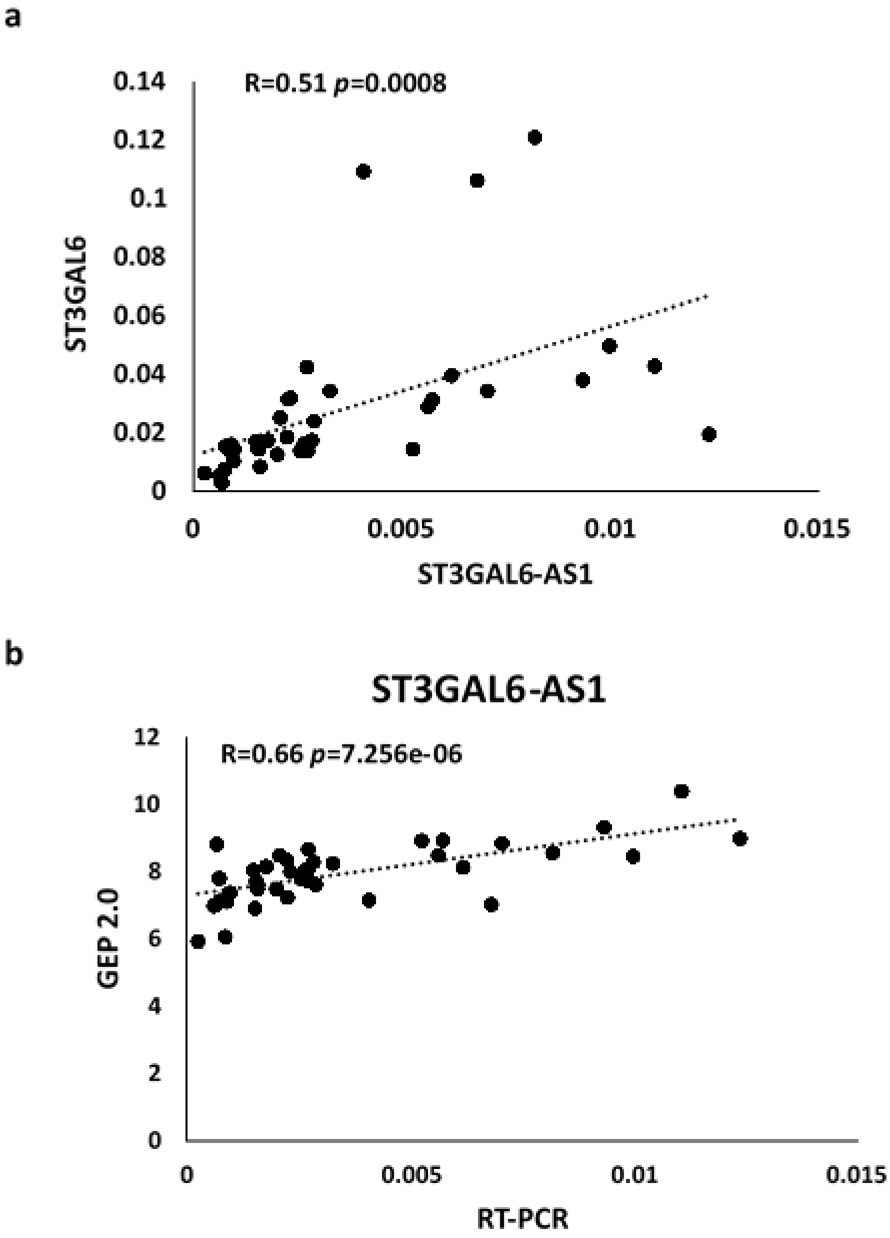

Figure S1. (**a**) Pearson's correlation on ST3GAL6-AS1 (x-axis) and *ST3GAL6* (y-axis) gene expression levels in 38 MM cases analyzed by qRT-PCR. (**b**) Pearson's correlation on ST3GAL6-AS1 transcript expression levels measured by qRT-PCR (x-axis) and microarray (y-axis). Correlation coefficients R and significant *p*-values are reported in both plots.

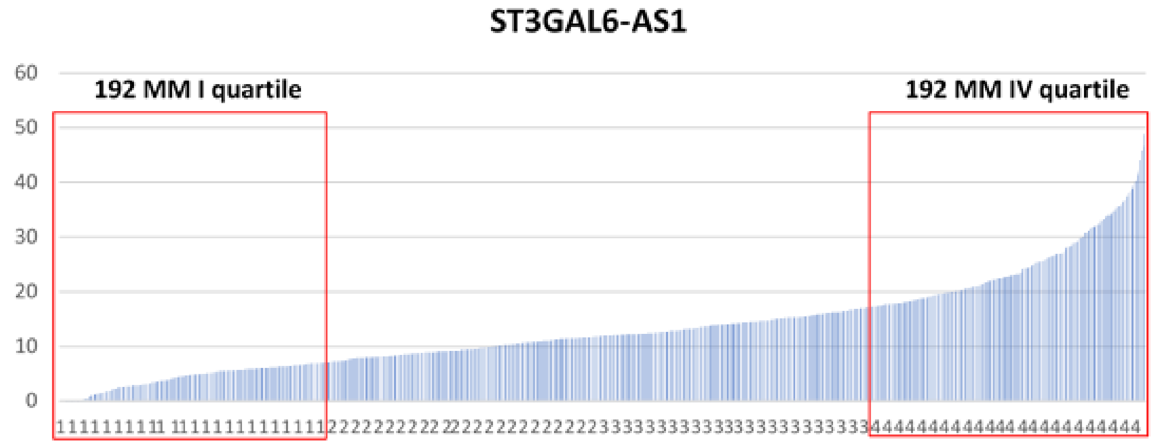

Figure S2. Histogram of ST3GAL6-AS1 expression level in 767 MM patients analyzed by RNA-sequencing in CoMMpass database. Samples are ordered according to increasing ST3GAL6-AS1 expression level and grouped into quartiles; 192 patients belonging to the two extreme quartiles are boxed in red.

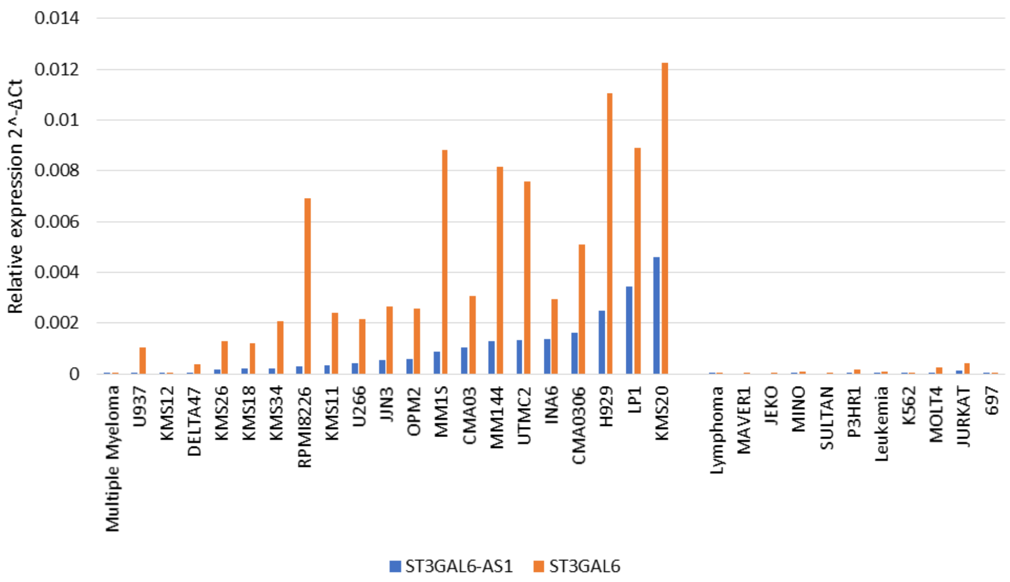

Figure S3. ST3GAL6-AS1 and *ST3GAL6* expression in hematological tumors including 21 HMCLs, 6 lymphomas (1 Diffuse Large B-Cell Lymphoma, 3 Mantle Cell Lymphomas, 2 B-Cell Lymphomas), and 5 leukemias (1 Chronic Lymphatic Leukemia, 1 Chronic Myeloid Leukemia, 2 T-cell Acute Lymphoblastic Leukemia, 1 B-Cell Acute Lymphoblastic Leukemia).

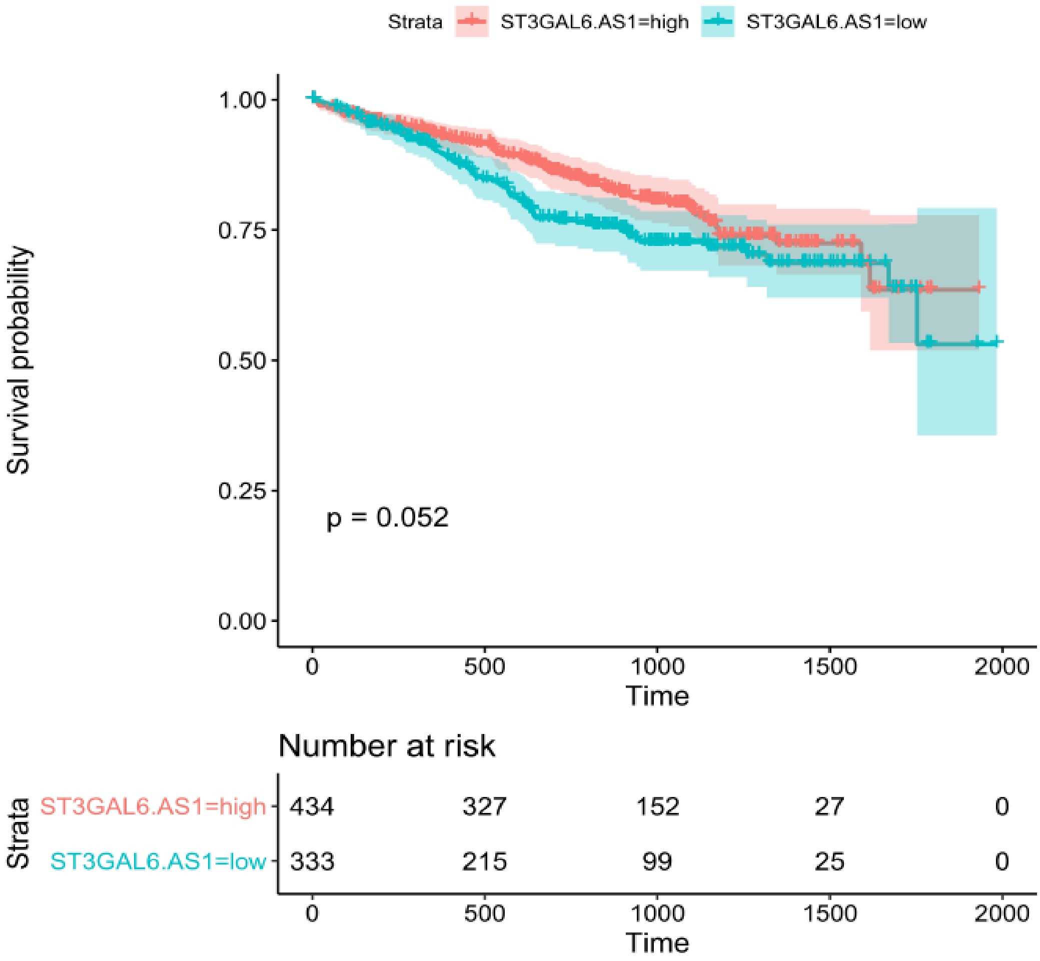

Figure S4. ST3GAL6-AS1 expression is unrelated to MM prognosis (OS) in CoMMpass database. Kaplan—Meier estimated curves of ST3GAL6-AS1 expression. MM are stratified into low or high-expressing groups. Confidence intervals are plotted. Log-rank *p*-value and a table risk with the absolute number of subjects at risk by time in each group are reported.

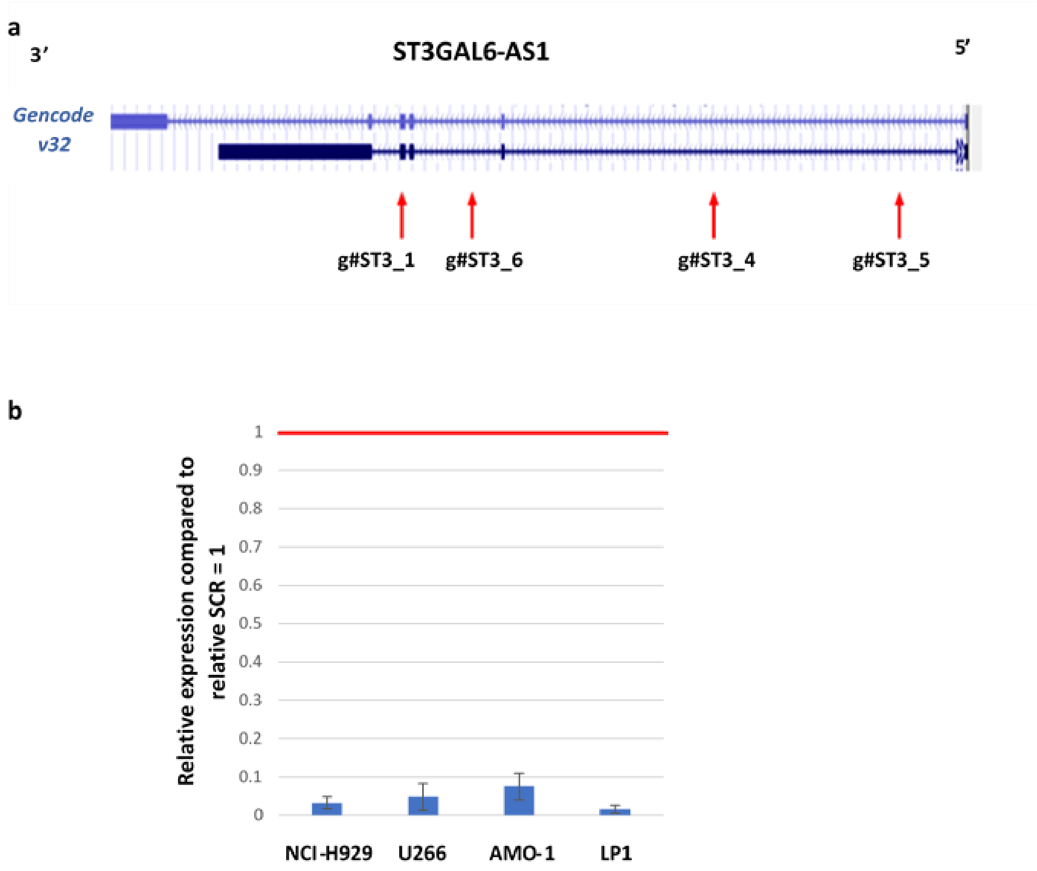

Figure S5. (**a**) Scheme of the 4 gapmeRs localization (Exiqon); (**b**) qRT-PCR analysis of ST3GAL6-AS1 silencing efficiency after 5 day from the gymnotic delivery of selected g#ST3_4 in the indicated HMCLs.

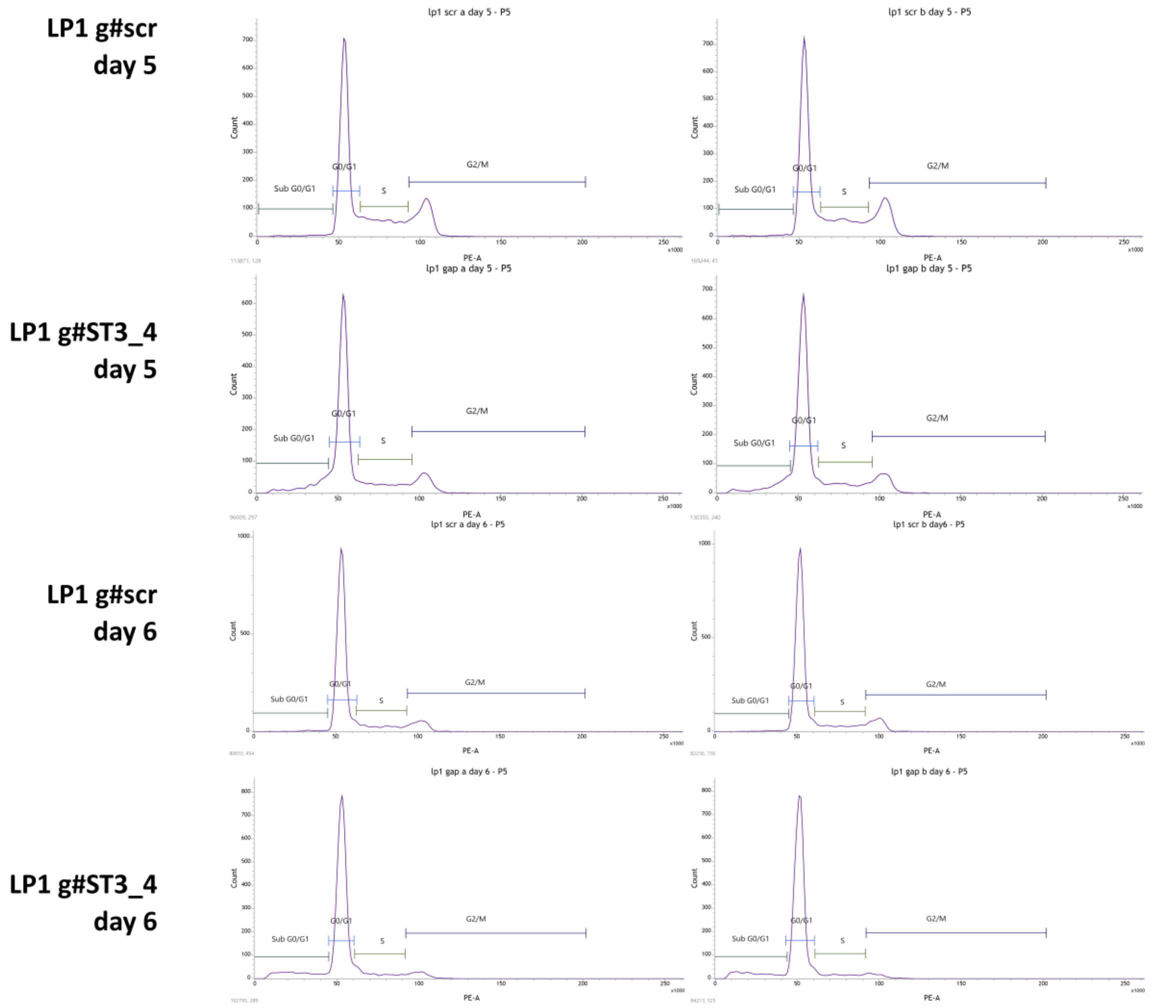

		sub G0/G1	G0/G1	S	G2/M
LP1 day 5	g#scr A	1.75	56.65	20.97	20.08
	g#scr B	1.34	57.42	20.65	19.99
	g#ST3_4 A	10.44	64.98	13.8	10.96
	g#ST3_4 B	9.97	65.63	13.75	10.87
LP1 day 6	g#scr A	1.14	78.49	11.19	9.22
	g#scr B	1.16	76.21	12.21	10.12
	g#ST3_4 A	12.06	72.35	9.75	5.55
	g#ST3_4 B	10.86	77.43	7.57	3.94

Figure S6. Cell cycle analysis by PI staining was performed in LP1 cells after treatment with g#ST3_4 gapmeR (5 μM).

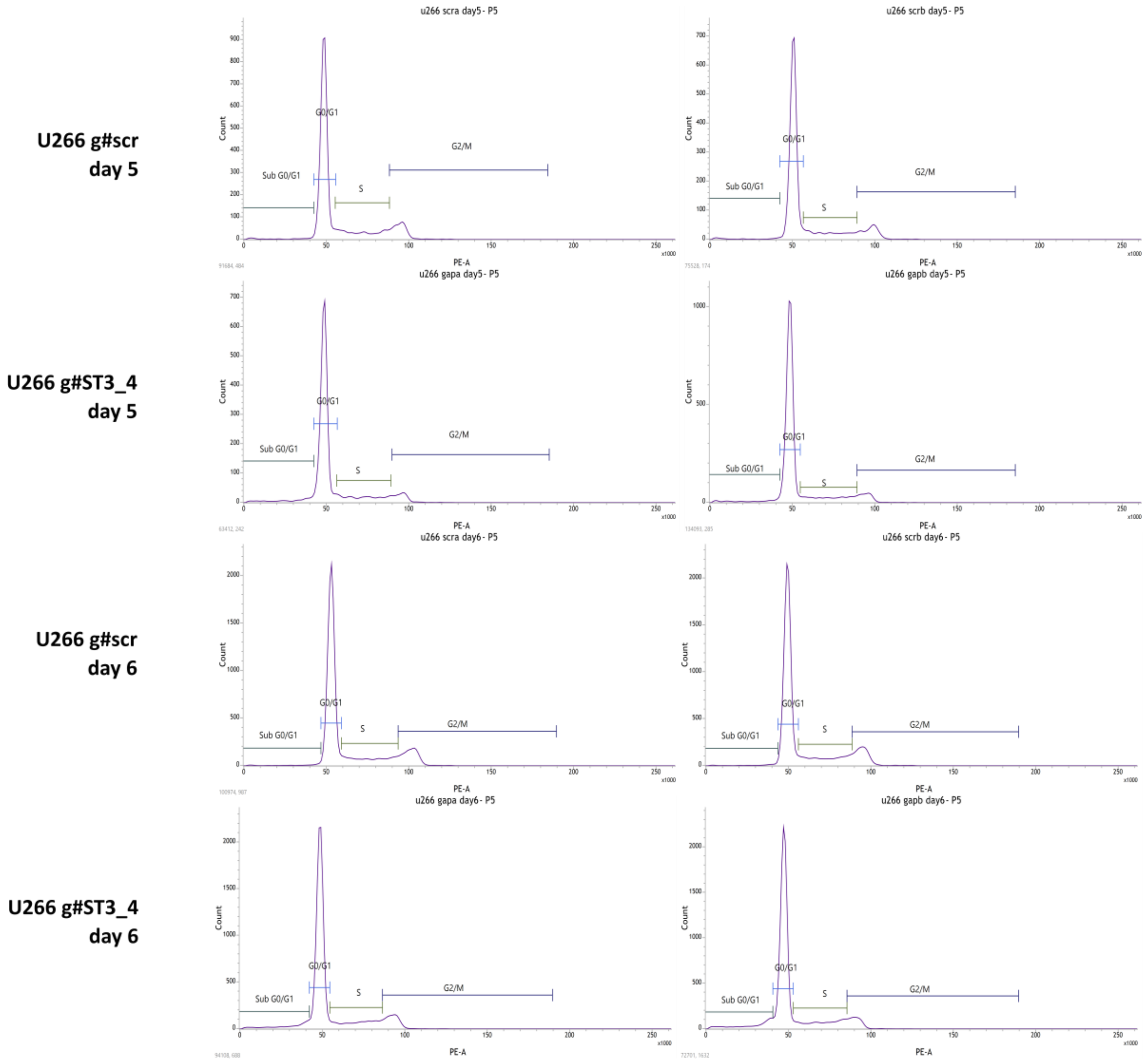

		sub G0/G1	G0/G1	S	G2/M
U266 day 5	g#scr A	1.7	69.15	16.93	12.4
	g#scr B	1.64	71.02	15.87	11.64
	g#ST3_4 A	5.96	73.39	14.56	6.88
	g#ST3_4 B	4.03	75.44	13.33	7.19
U266 day 6	g#scr A	1.43	69.14	15.53	13.44
	g#scr B	1.21	64.37	17.24	13.18
	g#ST3_4 A	6.33	69.39	14.1	10.18
	g#ST3_4 B	8.51	68.49	15.01	7.99

Figure S7. Cell cycle analysis by PI staining was performed in U266 cells after treatment with g#ST3_4 gapmeR (5 μM).

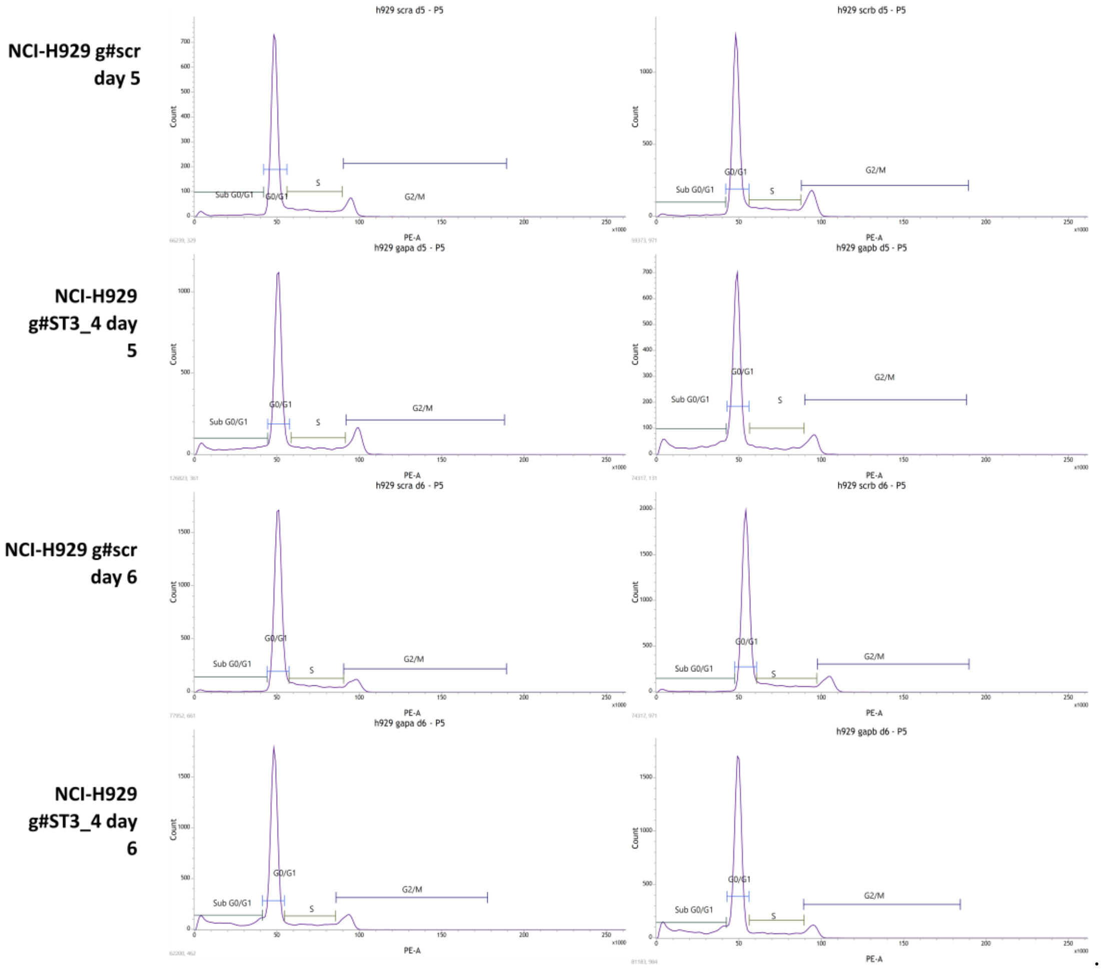

		sub G0/G1	G0/G1	S	G2/M
NCI-H929 day 5	g#scr A	6.04	68.17	16.08	9.18
	g#scr B	4.37	63.61	17.1	14.67
	g#ST3_4 A	16.02	58.3	12.83	12.14
	g#ST3_4 B	20.13	58.05	12.34	8.76
NCI-H929 day 6	g#scr A	1.99	71.14	16.25	10.04
	g#scr B	2.46	70.97	15.11	11.6
	g#ST3_4 A	18.15	62.94	10.43	8.86
	g#ST3_4 B	20.28	62.69	10.14	6.59

Figure S8. Cell cycle analysis by PI staining was performed in NCI-H929 cells after treatment with g#ST3_4 gapmeR (5 µM).

a

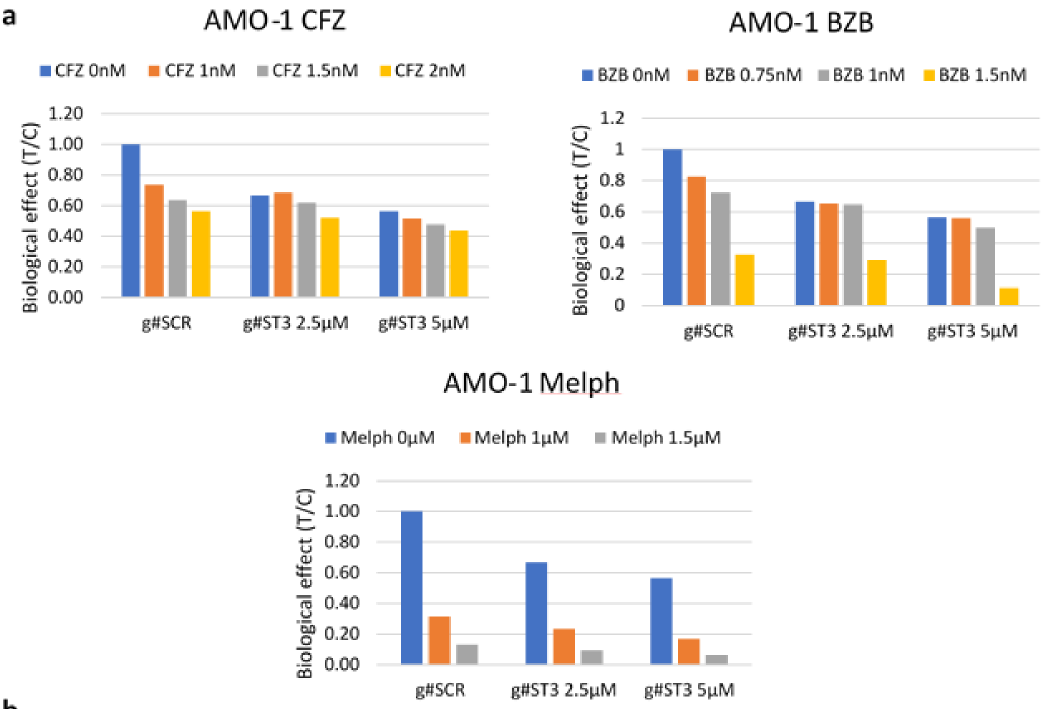

b

AMO-1_d6		g#ST3GAL6-AS1	
		2.5 µM	5 µM
Melph	1 µM	0.92	0.81
	1.5 µM	0.87	0.74
CFZ	1 nM	1.95	1.22
	1.5 nM	1.65	1.19
	2 nM	1.26	1.15
BZB	0.75 nM	1.6	1.65
	1 nM	1.85	1.5
	1.5 nM	1.01	0.67

		Additive			
1.45-1.20	1.20-1.10	1.10-0.90	0.90-0.85	0.85-0.70	0.70-0.30
Moderate	Slight		Slight	Moderate	Regular
Antagonism			Synergism		

Figure S9. ST3GAL6-AS1 KD antagonizes with CFZ or BZB, but not with Melph. (**a**) Raw-data of combination indexes (CI) resulting from combinatorial treatments of AMO-1 with g#ST3_4 and CFZ or Melph or BZB (6-day time point): fraction of viable AMO-1 cells after six days of treatment with g#ST3_4 (2,5 µM and 5 µM) and indicated concentrations of CFZ, Melph or BZB. (**b**) Table showing combination indexes (CI) resulting from combinatorial treatments of AMO-1 with g#ST3_4 gapmeR and CFZ, Melph, or BZB (6-day time point).

Figure 5e

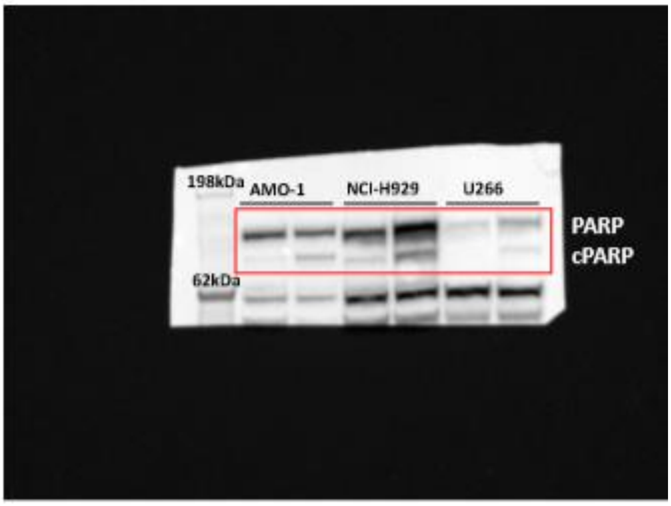

Figure 5e

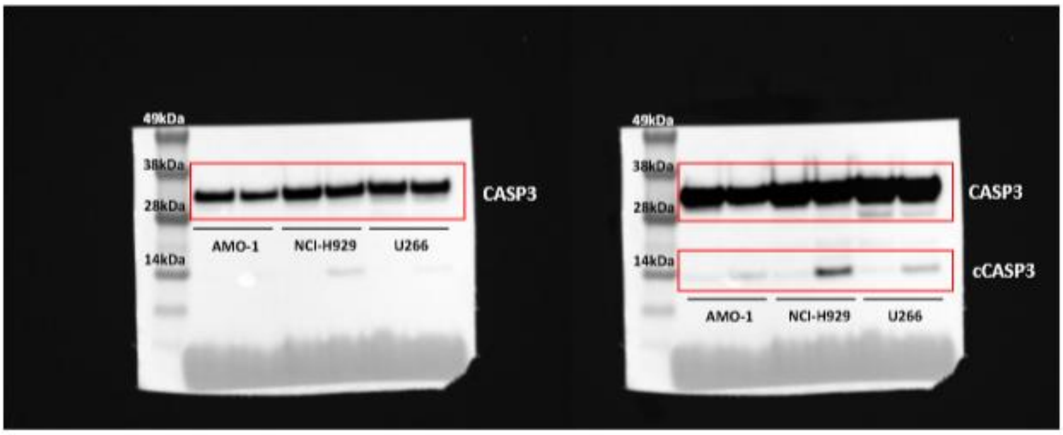

Figure 5e

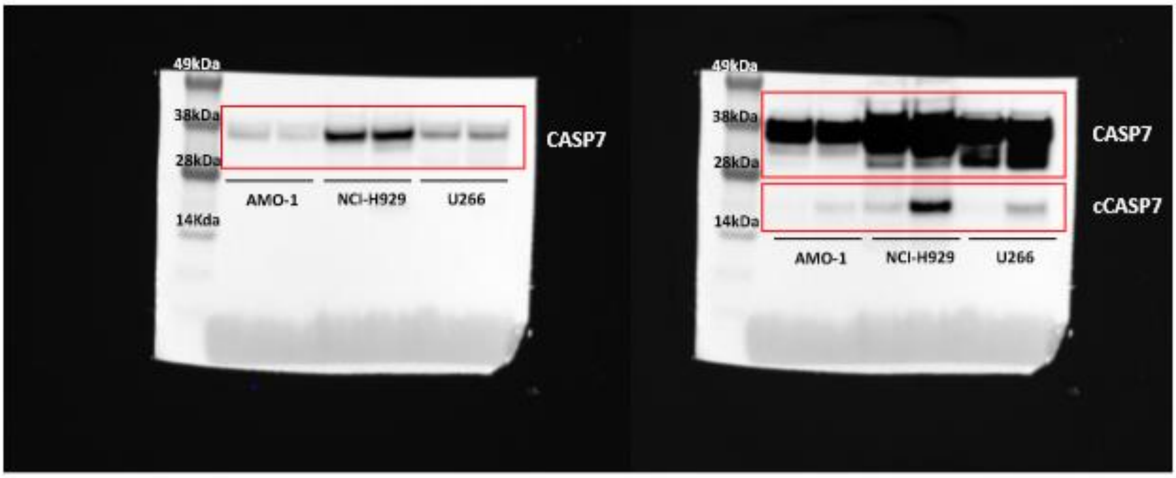

Figure 5e

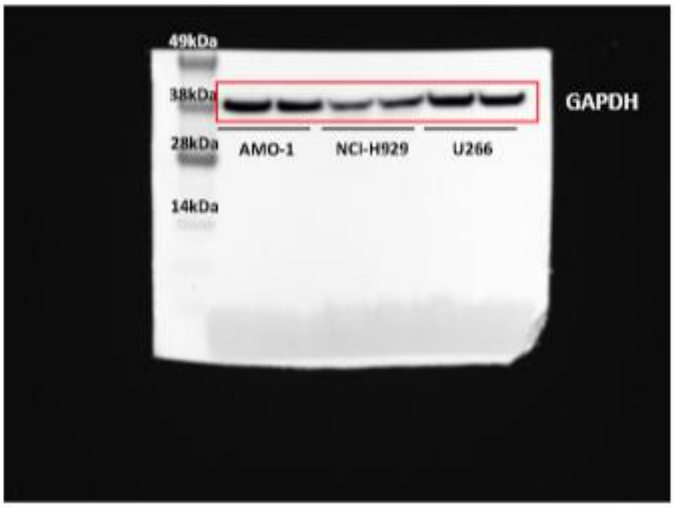

Figure 6b

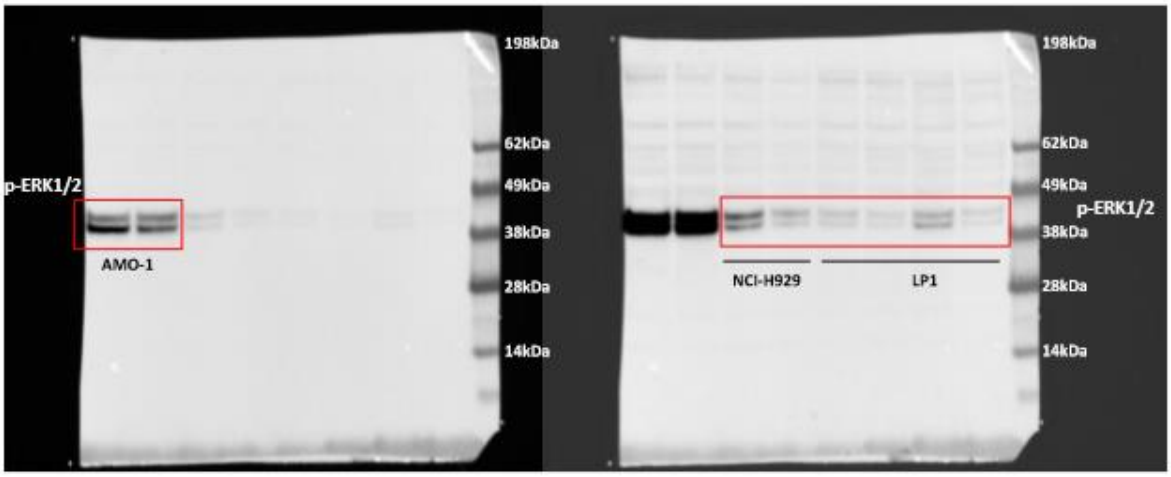

Figure 6b

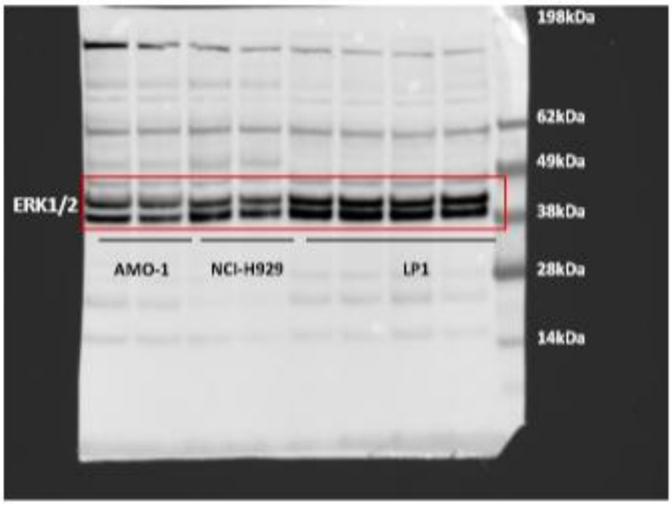

Figure 6b

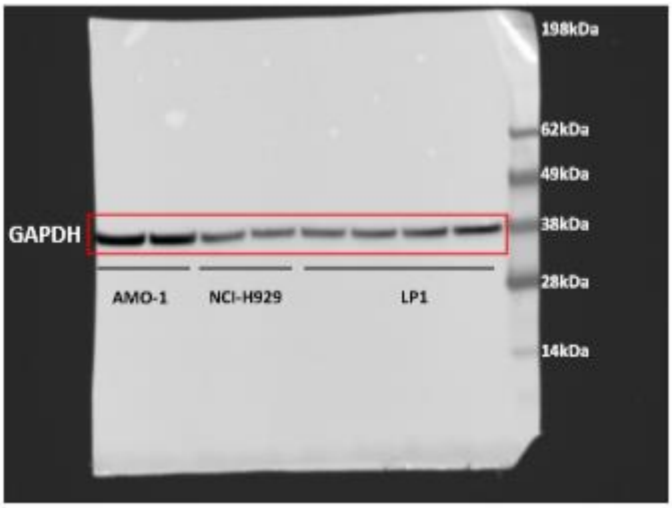

Figure 6b

Normalized densitometry readings and intensity ratio

		ERK1/2 normalized vs. GAPDH	p-ERK1/2 normalized vs. GAPDH	Ratio p-ERK1/2 ERK1/2	g#ST3_4/ g#SCR
AMO-1_d3	g#SCR	27016660	14170186	0.52449	1.000
	g#ST3_4	25344672	11910684	0.4699	0.896
NCI-H929_d3	g#SCR	56502334	20504672	0.36289	1.000
	g#ST3_4	55785738	7816788	0.1401	0.39
LP1_d3	g#SCR	86982299	5072524	0.05831	1.000
	g#ST3_4	90923724	2992040	0.03290	0.56
LP1_d4	g#SCR	71916321	8506932	0.1182	1.000
	g#ST3_4	61919065	2457126	0.03968	0.335

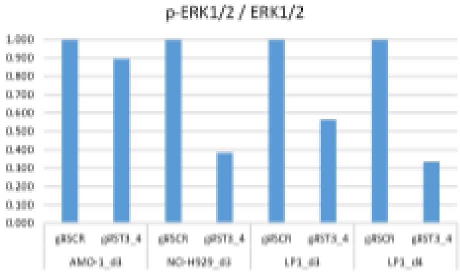

Figure 6d

UBIQUITIN

NCI-H929 U266 LP1 _ AMO-1

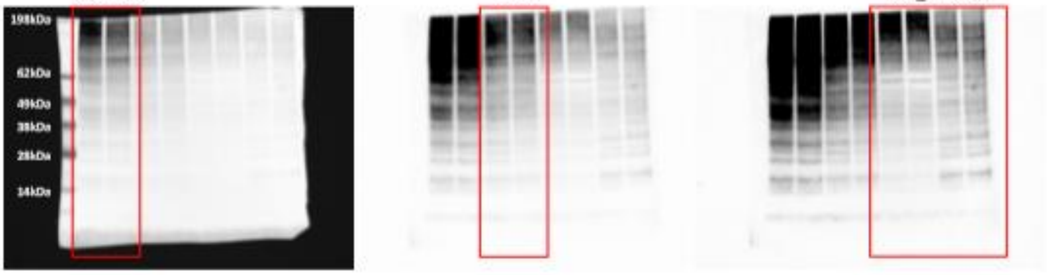

Figure 6d

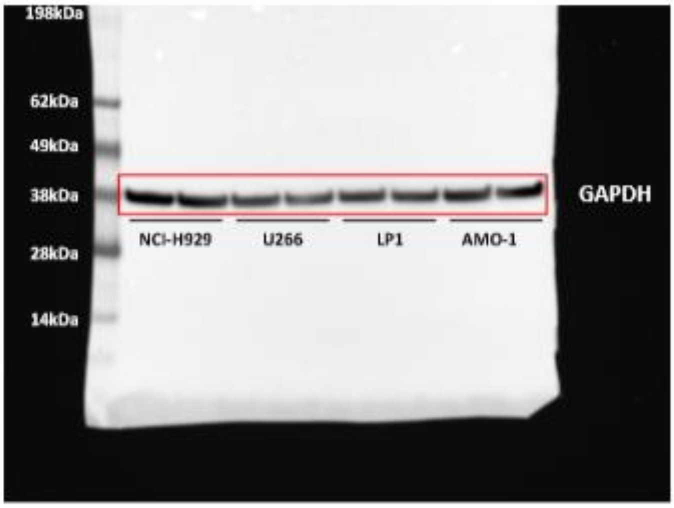

Figure 6d

Normalized densitometry readings and intensity ratio

		Ubiquitin (normalized vs. GAPDH)	g#ST3_4/g#SCR
NCI-H929	g#SCR	83955585	1
	g#ST3_4	51239520	0.610317
U266	g#SCR	92430730	1
	g#ST3_4	56206901	0.608098
LP1	g#SCR	81076240	1
	g#ST3_4	59712684	0.7365
AMO-1	g#SCR	21093167	1
	g#ST3_4	15875812	0.752652

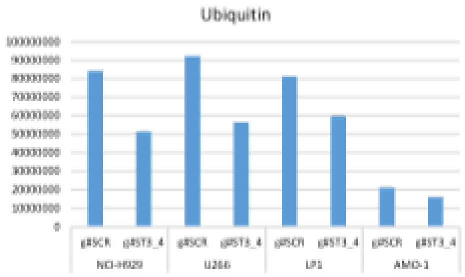

Figure 10. Uncropped western blots membrane acquisition showing all the bands with molecular weight markers for the indicated Figure and panel. Densitometry readings and intensity ratio of each band are provided for ubiquitin and ERK1/2 signal acquisition, respectively.

Table S1. Molecular characteristics of 50 MM patients.

Parameter	MM		
Median age, years (range)	67 (39–78)		
Male gender (%)	23 (46%)		
ISS, I–II–III	13–28–9		
**Sample Features**	**Positive (%)**	**Negative (%)**	**Not available**
HD	15 (30)	32 (64)	3
t(11;14)	12 (24)	38 (76)	0
t(4;14)	12 (24)	38 (76)	0
MAF-trx	5 (10)	45 (90)	0
none	3 (6)	47 (94)	
del(17p)	3 (6)	46 (92)	1
del(13q)	27 (54)	23 (46)	0
1q gain	24 (48)	20 (40)	6
N-RAS	8 (16)	33 (66)	9
K-RAS	12 (24)	29 (58)	9
BRAF	5 (10)	36 (72)	9
DIS3	9 (18)	32 (64)	9
P53	2(4)	38(76)	10
FAM46C	3(6)	36(72)	11

Table 2. List of the 31 differentially expressed lncRNAs identified by SAM (Significant Analysis of Microarray) analyses comparing plasma cells from MM patients with those from normal donors, ordered based on (d)-score.

Gene Name	Score(d)	Fold Change	Chromosome
ST3GAL6_AS1	6.04	7.98	3q12
AL035446_2	5.35	12.23	6q24
SNHG3	4.34	7.34	1p35
AC090517_5	4.23	2.92	15q21
AL355574_1	4.16	3.44	9q34
EPB41L4A_AS1	4.16	2.33	5q22
AC105339_2	4.14	4.29	15q25
AL035446_1	4.11	6.33	6q24
LINC01645	−7.99	0.44	1q25
AP003110_1	−7.58	0.33	11q14
AP002954_1	−6.72	0.39	11q23
AP000345_1	−5.73	0.27	22q11
WASIR2	−5.65	0.43	16p13
AC110995_1	−5.03	0.39	Xp22
MSC_AS1	−4.93	0.42	8q13
AL118511_2	−4.80	0.40	1q42
AC074135_1	−4.77	0.33	19p12
AC093865_1	−4.75	0.40	2q34
AP001189_1	−4.61	0.50	11q13
MME_AS1	−4.59	0.51	3q25
AL450344_3	−4.51	0.27	6q25
NCKAP5_AS2	−4.47	0.57	2q21
CXXC5_AS1	−4.37	0.48	5q31
AC035139_1	−4.37	0.27	10q11
MMP2_AS1	−4.34	0.53	16q12
AC103702_2	−4.34	0.40	17q21
LINC01222	−4.25	0.57	1q32
AL357033_2	−4.09	0.68	20q13
AC092849_2	−4.08	0.64	7q22
AC078878_2	−4.06	0.59	12q24
LINC01976	−3.99	0.63	17q21

Table S3. Number and relative frequency of main IgH translocations inferred from spike expression of the target genes in all 767 BM-1 MM cases analyzed by RNA sequencing; Major copy number alterations (CNAs) and non-synonymous somatic mutations in BM-1 MM cases for which CNAs and mutation counts data were also available by NGS-based FISH and Whole Exome sequencing (WES) analyses, respectively.

**767 BM_1 MM RNAseq**	
**IgH trx**	***N* (Percent)**
t(11;14)/CCND1	153 (20%)
t(6;14)/CCND3	11 (1%)
t(4;14)/WHSC1/FGFR3	106 (14%)
t(14;16)/MAF;t(14;20)/MAFB; t(8;16)/MAFA	52 (7%)
t(8;14)/MYC	37 (5%)
**656 BM_1 MM RNAseq-WES.CNA**	
**CNA**	***N* (Percent)**
1q21 gain	218 (33%)
del(13)(q14)/(q34)/RB1 biallelic loss	317 (48%)
del(17)(p13)/TP53 biallelic loss	52 (8%)
del(1)(p22)/CDKN2C biallelic loss	162 (25%)
**724 BM_1 MM RNAseq-WES.MUT ***	
**Non syn. Somatic mutation**	***N* (Percent)**
1–3 KRAS mut	179 (25%)
1–2 NRAS mut	158 (22%)
1–2 BRAF mut	57 (8%)
HRAS mut	0 (0%)
1–3 DIS3 mut	77 (11%)
1–3 FAM46C mut	72 (10%)
1–3 TP53 mut	32 (4%)

* WES-MUT refers to non-synonymous somatic mutation counts data; the number of possible mutations in each gene for each MM patient is reported.

Table S4. Number and relative frequency of main IgH translocations, CNAs and non-synonymous somatic mutations in BM-1 MM cases of ST3GAL6-AS1 quartile I and IV, respectively.

**Genetic Abnormality**	**192 BM_1 MM ST3GAL6_AS1 Quartile I**	**192 BM_1 MM ST3GAL6_AS1 Quartile IV**
**IgH trx**	***N* (Percent)**	***N* (Percent)**
t(11;14)/CCND1	37 (19%)	43 (22%)
t(6;14)/CCND3	4 (2%)	3 (2%)
t(4;14)/WHSC1/FGFR3	29 (15%)	21 (11%)
t(14;16)/MAF;t(14;20)/MAFB; t(8;16)/MAFA	12 (6%)	17 (9%)
t(8;14)/MYC	14 (7%)	6 (3%)
**CNA**	**164 BM_1 MM ST3GAL6_AS1 Quartile I *N* (Percent)**	**164 BM_1 MM ST3GAL6_AS1 Quartile IV *N* (Percent)**
1q21 gain	48 (29%)	68 (41%)
del(13)(q14)/(q34)/RB1 biallelic loss	76 (46%)	86 (52%)
del(17)(p13)/TP53 biallelic loss	12 (7%)	12 (7%)
del(1)(p22)/CDKN2C biallelic loss	44 (27%)	34 (21%)
**Non syn. Somatic mutation**	**181 BM_1 MM ST3GAL6_AS1 Quartile I *N* (Percent)**	**181 BM_1 MM ST3GAL6_AS1 Quartile IV *N* (Percent)**
1-3 KRAS mut	38 (21%)	46 (25%)
1-2 NRAS mut	24 (13%)	53 (29%)
1-2 BRAF mut	12 (7%)	20 (11%)
HRAS mut	0 (0%)	0 (0%)
1-3 DIS3 mut	10 (6%)	27 (15%)
1-3 FAM46C mut	17 (9%)	17 (9%)
1-3 TP53 mut	9 (5%)	8 (4%)

Table S5. List of the significantly enriched Annotation Clusters for the 482 differentially expressed protein coding genes (Enrichment Score >2) by DAVID 6.8 Bioinformatics tool.

Annotation Cluster	**Enrichment Score**	**GO Term**	**Count**	**%**	***p* Value**	**Genes**
**Annotation Cluster 1**	**9.521**	**GO:0016477~cell migration**	68	14.81	4.92E-11	CTHRC1, DMTN, INSL3, NRG3, PTGS2, S100A8, PREX1, FAM110C, STAT5B, S100A9, WWC1, CXCR1, CXCR2, PRKG1, GDNF, GLI3, TNFRSF11A, CD44, CXCR5, CCL3L1, PAK3, SEMA7A, RAB25, PDGFD, FAM83H, EGR3, VANGL2, ARTN, PADI2, SIX2, PPARGC1A, NDNF, SLIT2, HES1, RETN, BVES, RRAS2, DLL4, FOXC1, TREM1, CTSH, CXCL1, GPR183, CCL3, FUT7, CXCL3, COL3A1, CXCL2, CDH2, ITGAM, CCL25, ZNF703, SFTPD, DCLK1, BMP4, IL6, SLC8A1, ABR, S100P, ANXA1, MCAM, ANXA3, S100A12, CHRD, MYLK, PLAU, SRMS, LRP5
**Annotation Cluster 2**	**7.121**	**GO:0007417~central nervous system development**	51	11.11	3.97E-08	SYT1, HNF1B, NRG3, S100A8, S100A9, BCAN, PAX5, CXCR2, PRKG1, GLI3, CASP5, APP, SERPINE2, SEMA7A, GRID2, VNN2, PITX1, FA2H, BCL2A1, PCDH9, PADI2, BASP1, PPARGC1A, SLIT2, HES1, SSTR3, DLL4, CNTN1, FOXC1, COL3A1, CDH2, NDRG2, DCLK1, CD27, BMP4, NES, SLC8A1, ABR, GABRA4, ARMC4, MAL, FZD4, PPP1R17, ANXA3, TP73, FZD6, DKK1, ITGA8, LRP6, AVPR1A, CHRD
**Annotation Cluster 3**	**6.747**	**GO:0030595~leukocyte chemotaxis**	21	4.58	5.31E-08	CXCL1, GPR183, IL6, CCL3, S100A8, CXCL3, PREX1, CXCL2, S100A9, ANXA1, CXCR1, PADI2, CXCR2, SLIT2, S100A12, CCL25, TNFRSF11A, CXCR5, CCL3L1, SFTPD, TREM1
**Annotation Cluster 4**	**6.559**	**GO:0010646~regulation of cell communication**	117	25.49	8.10E-08	SYT1, CTHRC1, S100A8, PTGS2, FGF14, FAM110C, S100A9, PMAIP1, GDNF, TLR9, APP, DIRAS2, CD44, SERPINE2, SEMA7A, PDGFD, SPRED1, CRY1, HCAR2, SOCS3, VANGL2, FBP1, SIPA1L2, PPARGC1A, HCST, HES1, RETN, RASGRF1, DLL4, DBN1, CAV2, IL1R2, CCL3, BLK, AKAP12, KCNA5, KCNS3, RNF165, BCHE, NDRG2, RAP1GAP2, ARHGEF10L, BMP4, ABR, TRIO, PTPN13, S100A12, ITGA8, AVPR1A, DEPTOR, CHRD, SRMS, IFI6, PLAU, IER3, DMTN, SH3RF1, HNF1B, LY86, PREX1, WWC1, GLI3, ZNF205, MCF2L, USP18, TNFRSF11A, PAK3, CCL3L1, HEY2, SPG20, NRG2, NOS1AP, PADI2, TLE1, GZMB, ARHGAP24, SLIT2, SIGIRR, DAPK1, PROK2, GRM4, MYRIP, G0S2, CTSH, PMEPA1, GPR183, COL3A1, CDH2, TSPYL5, RGS10, CCL25, ZNF703, PLEKHG1, LRG1, ARHGAP44, LEFTY2, BCL6, NFATC4, CD27, SLC8A1, IL6, FLT3, ANXA1, NLGN1, RGS18, MAL, FZD4, FZD7, TP73, FZD6, PLEKHF1, PPP1R9A, CD19, DKK1, RASSF2, LRP6, LRP5
**Annotation Cluster 5**	**5.749**	**GO:0030593~neutrophil chemotaxis**	13	2.83	8.05E-07	CXCL1, CCL25, CCL3, S100A8, CCL3L1, PREX1, CXCL3, CXCL2, S100A9, CXCR2, TREM1, SLIT2, S100A12

**Annotation Cluster 6**	**5.249**	**GO:0051270~regulation of cellular component movement**	43	9.37	2.17E-06	CXCL1, GPR183, INSL3, DMTN, CCL3, NRG3, PTGS2, CXCL3, FAM110C, CXCL2, COL3A1, CXCR2, FES, CCL25, ZNF703, SERPINE2, PAK3, SEMA7A, BCL6, RAB25, PDGFD, FAM83H, NRG2, BMP4, SLC8A1, IL6, ABR, NOS1AP, ANXA1, ARMC4, PADI2, MCAM, PPARGC1A, ANXA3, SLIT2, CTNNA3, RETN, RRAS2, DLL4, CTSH, CHRD, PLAU, MYLK
**Annotation Cluster 7**	**5.184**	**GO:0012501~programmed cell death**	77	16.78	2.53E-06	IER3, SH3RF1, HNF1B, PTGS2, GABRB3, S100A8, LY86, STAT5B, S100A9, CXCR2, PMAIP1, GDNF, GLI3, ZNF205, MCF2L, ASAH2, CASP5, APP, TNFRSF11A, CD44, PAK3, HEY2, GRID2, HCAR2, EGR3, NOS1AP, SOCS3, BCL2A1, CECR2, GZMB, TLE1, GZMH, PPARGC1A, NDNF, SLIT2, DAPK1, PROK2, GRM4, SSTR3, SCIN, MNDA, FOXC1, G0S2, CTSH, CCL3, BLK, HK2, CD70, APLP1, LEFTY2, BCL6, NFATC4, NDRG1, XAF1, CD27, BMP4, NES, CRIP1, IL6, ABR, FLT3, ANXA1, TRIO, GAS2, MAL, TNFSF9, TP73, TNFSF8, IFIT3, PLEKHF1, LCN2, IFI27, RASSF2, LRP6, DEPTOR, IFI6, LRP5
**Annotation Cluster 8**	**4.718**	**GO:0040017~positive regulation of locomotion**	28	6.10	4.61E-06	CXCL1, INSL3, DMTN, CCL3, PTGS2, CXCL3, FAM110C, CXCL2, CXCR2, ZNF703, PAK3, SEMA7A, RAB25, PDGFD, FAM83H, BMP4, SLC8A1, IL6, ARTN, MCAM, SLIT2, ANXA3, RETN, BVES, RRAS2, CTSH, MYLK, PLAU
**Annotation Cluster 9**	**4.390**	**GO:0001822~kidney development**	20	4.36	2.90E-05	BMP4, GCNT4, HNF1B, VANGL2, SIX2, MME, CXCR2, BASP1, GDNF, GLI3, TP73, SLIT2, HES1, OSR2, HOXB7, ITGA8, OVOL1, FOXC1, PDGFD, CTSH
**Annotation Cluster 10**	**4.295**	**GO:0023056~positive regulation of signaling**	65	14.16	1.95E-05	DMTN, SYT1, PTGS2, S100A8, LY86, FAM110C, S100A9, WWC1, PMAIP1, ZNF205, MCF2L, TLR9, DIRAS2, TNFRSF11A, CD44, SERPINE2, CCL3L1, PAK3, SEMA7A, SPRED1, PDGFD, HCAR2, NOS1AP, SOCS3, VANGL2, GZMB, HCST, HES1, RETN, PROK2, GRM4, MYRIP, BVES, RASGRF1, DLL4, G0S2, CTSH, CAV2, GPR183, CCL3, BLK, COL3A1, AKAP12, CDH2, TSPYL5, CCL25, LRG1, RNF165, LEFTY2, NFATC4, CD27, BMP4, IL6, FLT3, NLGN1, MAL, FZD4, TP73, FZD7, S100A12, PLEKHF1, PPP1R9A, ITGA8, RASSF2, LRP6
**Annotation Cluster 11**	**4.020**	**GO:0043065~positive regulation of apoptotic process**	31	6.75	6.37E-05	CCL3, S100A8, PTGS2, S100A9, CXCR2, PMAIP1, ZNF205, MCF2L, PAK3, BCL6, NFATC4, HCAR2, BMP4, IL6, ABR, BCL2A1, ANXA1, TRIO, MAL, GZMB, PPARGC1A, SLIT2, TP73, DAPK1, PLEKHF1, IFI27, RASSF2, SCIN, MNDA, G0S2, CTSH
**Annotation Cluster 12**	**3.817**	**GO:0070486~leukocyte aggregation**	28	6.10	1.74E-05	HLA-DQB1, GPR183, S100A8, LRRC32, ELF4, FUT7, PREX1, STAT5B, S100A9, GLI3, BATF, CD44, PAK3, SFTPD, BCL6, CD27, BMP4, EGR3, IL6, ANXA1, CD1C, TNFSF9, FZD7, TNFSF8, CD1D, HES1, DLL4, HLA-DRA
**Annotation Cluster 13**	**3.625**	**GO:0001568~blood vessel development**	30	6.54	1.66E-04	PTGS2, COL3A1, CXCR2, CDH2, GLI3, LRG1, HEY2, NFATC4, PDGFD, BMP4, EGR3, COL4A2, SOCS3, ARHGAP24, MCAM, FZD4, SLIT2, NDNF, ANXA3, HES1, PROK2, CYBB, DLL4, ZMIZ1, FOXC1, ANTXR1, CTSH, TNFAIP2, MYLK, LRP5

**Annotation Cluster 14**	**3.486**	**GO:0001944~vasculature development**	31	6.75	1.99E-04	PTGS2, COL3A1, CXCR2, CDH2, GLI3, LRG1, HEY2, NFATC4, PDGFD, BMP4, IL6, EGR3, COL4A2, SOCS3, ARHGAP24, MCAM, FZD4, SLIT2, NDNF, ANXA3, HES1, PROK2, CYBB, DLL4, ZMIZ1, FOXC1, ANTXR1, CTSH, TNFAIP2, MYLK, LRP5
**Annotation Cluster 15**	**3.069**	**GO:0022008~neurogenesis**	58	12.64	1.66E-04	CTHRC1, SYT1, NRG3, GABRB3, S100A8, GRIP1, PREX1, S100A9, NAP1L2, FES, PRKG1, GLI3, GDNF, TLR9, APP, SERPINE2, PAK3, SEMA7A, HEY2, GRID2, SPG20, FA2H, VANGL2, ARTN, CECR2, NDNF, SLIT2, HES1, NYAP2, RASGRF1, DLL4, CNTN1, DBN1, GPR183, COBL, CCL3, COL3A1, CDH2, BCHE, RNF165, ETV1, BCL6, NFATC4, NDRG1, EHD1, RAP1GAP2, DCLK1, BMP4, IL6, NLGN1, ANXA1, ZNF521, FZD4, TP73, FZD7, PPP1R9A, DKK1, LRP6
**Annotation Cluster 16**	**2.997**	**GO:0060562~epithelial tube morphogenesis**	19	4.14	5.58E-04	BMP4, CTHRC1, COBL, HNF1B, VANGL2, CECR2, CXCR2, GDNF, GLI3, SLIT2, FZD6, HES1, HOXB7, DLL4, ST14, LRP6, NFATC4, CTSH, LRP5
**Annotation Cluster 17**	**2.937**	**GO:0009952~anterior/posterior pattern specification**	16	3.49	1.10E-04	BMP4, MYF6, COBL, HNF1B, VANGL2, SIX2, BASP1, GLI3, HES1, DKK1, HOXB7, HEY2, HOXA10, LRP6, FOXC1, LRP5
**Annotation Cluster 18**	**2.897**	**GO:0050767~regulation of neurogenesis**	32	6.97	5.99E-04	SYT1, COBL, GPR183, NRG3, PREX1, COL3A1, CDH2, NAP1L2, FES, GLI3, APP, SERPINE2, PAK3, SEMA7A, HEY2, SPG20, GRID2, BCL6, NFATC4, RAP1GAP2, BMP4, IL6, NLGN1, TP73, SLIT2, NDNF, HES1, PPP1R9A, DKK1, DLL4, CNTN1, DBN1
**Annotation Cluster 19**	**2.817**	**GO:0001823~mesonephros development**	11	2.40	1.96E-04	HES1, BMP4, HNF1B, OSR2, HOXB7, SIX2, FOXC1, BASP1, GLI3, GDNF, SLIT2
**Annotation Cluster 20**	**2.735**	**GO:0009893~positive regulation of metabolic process**	99	21.57	0.0014	S100A8, PTGS2, S100A9, STAT5B, PMAIP1, GDNF, SOHLH2, TLR9, BATF, APP, DIRAS2, CD44, SEMA7A, PDGFD, PITX1, SOCS3, VANGL2, SIX2, PPARGC1A, HES1, RETN, KRT17, DLL4, SNX9, CAV2, CCL3, ZNF516, FCER2, AKAP12, MEIS2, HOXA10, AUTS2, ARHGEF10L, BMP4, TESC, IKZF2, S100A12, LCN2, IFI27, ITGA8, AVPR1A, ANTXR1, HNF1B, NRG3, GRIP1, ELF4, WWC1, PAX5, NAP1L2, GLI3, ZNF205, TNFRSF11A, OSR2, CCL3L1, PAK3, HEY2, GUCY1A2, NOS1AP, PADI2, TLE1, ARRDC4, DAPK1, PROK2, GRM4, BVES, ZMIZ1, CAND2, CNTN1, FOXC1, CTSH, GLIS3, GPR183, TYRP1, LRRC32, CDH2, TSPYL5, CCL25, ACSL1, LEFTY2, RNF128, ETV1, BCL6, NFATC4, CD27, MYF6, CR1, IL6, FLT3, ANXA1, FZD4, ANXA3, TP73, FZD7, TNFSF8, PPP1R9A, CYBB, RASSF2, LRP6, LRP5
**Annotation Cluster 21**	**2.709**	**GO:0009617~response to bacterium**	29	6.32	1.53E-04	ALPL, CXCL1, CCL3, S100A8, PTGS2, CXCL3, LY86, CXCL2, S100A9, ACP5, TLR9, APP, TNFRSF11A, SFTPD, CD27, COCH, IL6, ABR, PPARGC1A, ANXA3, SIGIRR, TNFSF8, CD1D, S100A12, LCN2, TJP1, DEFA4, SLPI, TREM1

**Annotation Cluster 22**	**2.707**	**GO:0023014~signal transduction by protein phosphorylation**	37	8.06	0.0014	SH3RF1, GPR183, CAV2, CCL3, FGF14, WWC1, CDH2, GDNF, TLR9, CCL25, TNFRSF11A, DIRAS2, CD44, CCL3L1, PAK3, SEMA7A, STK39, SPRED1, NDRG2, PDGFD, NRG2, CD27, BMP4, IL6, FLT3, VANGL2, ARTN, FZD4, TP73, FZD7, S100A12, PROK2, GRM4, RASGRF1, RASSF2, MAP3K19, CTSH
**Annotation Cluster 23**	**2.597**	**GO:0048534~hematopoietic or lymphoid organ development**	34	7.41	0.0013	DMTN, GPR183, CCL3, FUT7, PREX1, STAT5B, FES, GLI3, ITM2A, BATF, TNFRSF11A, MEIS2, CXCR5, BCL6, CD27, BMP4, IL6, TESC, EGR3, FLT3, ANXA1, ARTN, TNFSF9, FZD7, CD1D, TNFSF8, HES1, BVES, HOXB7, DLL4, SCIN, RASSF2, FOXC1, LRP5
**Annotation Cluster 24**	**2.524**	**GO:0061217~regulation of mesonephros development**	6	1.31	3.50E-04	BMP4, HNF1B, HOXB7, SIX2, BASP1, GDNF
**Annotation Cluster 25**	**2.424**	**GO:0060993~kidney morphogenesis**	10	2.18	4.52E-04	HES1, BMP4, GCNT4, HNF1B, HOXB7, VANGL2, SIX2, BASP1, GLI3, GDNF
**Annotation Cluster 26**	**2.412**	**GO:0060113~inner ear receptor cell differentiation**	7	1.53	0.0032	HES1, BMP4, CTHRC1, GABRB3, HEY2, VANGL2, CECR2
**Annotation Cluster 27**	**2.298**	**GO:0032270~positive regulation of cellular protein metabolic process**	52	11.33	0.0028	NRG3, S100A8, S100A9, WWC1, PMAIP1, NAP1L2, TLR9, APP, TNFRSF11A, DIRAS2, CD44, PAK3, CCL3L1, SEMA7A, PDGFD, NOS1AP, SOCS3, VANGL2, ARRDC4, PPARGC1A, DAPK1, HES1, PROK2, GRM4, KRT17, CNTN1, CTSH, SNX9, GPR183, CAV2, CCL3, CDH2, TSPYL5, CCL25, ACSL1, LEFTY2, RNF128, AUTS2, BCL6, CD27, BMP4, CR1, IL6, FLT3, FZD4, FZD7, TP73, S100A12, IFI27, PPP1R9A, RASSF2, ANTXR1
**Annotation Cluster 28**	**2.279**	**GO:0019220~regulation of phosphate metabolic process**	57	12.42	0.0035	DMTN, SH3RF1, NRG3, GABBR1, WWC1, TLR9, APP, DIRAS2, TNFRSF11A, CD44, CCL3L1, PAK3, SEMA7A, GUCY1A2, STK39, SPRED1, PDGFD, DLG2, SOCS3, VANGL2, FBP1, PPARGC1A, SLIT2, HES1, PROK2, GRM4, CNTN1, PMEPA1, ME1, SNX9, GPR183, CAV2, CCL3, AKAP12, CDH2, APLP1, CCL25, ACSL1, LEFTY2, CDA, NDRG2, CD27, BMP4, TESC, IL6, FLT3, FZD4, TP73, PPP1R17, FZD7, S100A12, PPP1R9A, DKK1, RASSF2, LRP6, DEPTOR, LRP5
**Annotation Cluster 29**	**2.231**	**GO:0001781~neutrophil apoptotic process**	4	0.87	2.67E-04	IL6, ANXA1, CXCR2, HCAR2

**Annotation Cluster 30**	**2.214**	**GO:0010648~negative regulation of cell communication**	44	9.59	0.0048	IL1R2, CAV2, SH3RF1, CTHRC1, PTGS2, WWC1, CDH2, GLI3, GDNF, TLR9, RGS10, CD44, SERPINE2, BCHE, HEY2, SPG20, BCL6, NFATC4, SPRED1, NDRG2, CRY1, BMP4, IL6, SOCS3, FBP1, PADI2, TLE1, RGS18, TP73, SLIT2, DAPK1, SIGIRR, FZD6, PPP1R9A, DKK1, DLL4, RASSF2, LRP6, AVPR1A, DEPTOR, CHRD, PMEPA1, IFI6, SRMS
**Annotation Cluster 31**	**2.172**	**GO:0001838~embryonic epithelial tube formation**	10	2.18	0.0035	BMP4, CTHRC1, COBL, HNF1B, VANGL2, ST14, LRP6, CECR2, GDNF, FZD6
**Annotation Cluster 32**	**2.074**	**GO:0043410~positive regulation of MAPK cascade**	26	5.66	3.37E-04	GPR183, CAV2, CCL3, WWC1, CDH2, TLR9, CCL25, TNFRSF11A, DIRAS2, CD44, PAK3, CCL3L1, SEMA7A, PDGFD, CD27, BMP4, IL6, FLT3, VANGL2, FZD4, FZD7, TP73, S100A12, PROK2, GRM4, RASSF2
**Annotation Cluster 33**	**2.069**	**GO:0010720~positive regulation of cell development**	24	5.23	0.0016	BMP4, MYF6, COBL, SYT1, DMTN, IL6, PREX1, NLGN1, NAP1L2, FES, GDNF, GLI3, NDNF, SLIT2, TP73, HES1, PPP1R9A, ZNF703, DKK1, SERPINE2, PAK3, SEMA7A, CNTN1, BCL6

Table S6. KEGG gene sets obtained using Gene Sets Enrichment Analysis showing significant modulation between ST3GAL6-AS1 IV Quartile and ST3GAL6-AS1 I Quartile in CoMMpass database. (NES >1.5 or <-1.5; *p*-value <0.05, NES: Normalized Enrichment Score).

NAME	SIZE	NES	NOM *p*-val	Gene List *
**KEGG_UBIQUITIN_MEDIATED_PROTEOLYSIS**	130	1.866	0.0020	**UBE2E1, UBA3, UBE2E3, ANAPC13, TRIM32, UBE2W, CDC23, PIAS1, ITCH, SKP2, UBE2E2, KLHL9, UBE3B, UBE2K, SKP1, ERCC8, FANCL, UBE2J1, PIAS3, ANAPC7, UBE3A, PIAS2, FBXW11, UBE3C, CUL2, RCHY1, UBE4B, UBA2, UBE2N, UBE2B, HERC4, DET1, BIRC6, UBE2D2, FBXO4, UBE2Q2, NHLRC1, PML, UBA6, UBE2Q1, VHL, RBX1, BTRC, ANAPC10, WWP1, RNF7, ANAPC4, UBE2D4, CUL1, TRIP12, UBE2R2,** PPIL2, ANAPC1, UBE2I, STUB1, WWP2, TRIM37, UBE2QL1, HERC1, SAE1, AIRE, UBE2H, UBE2Z, UBE2J2, ANAPC2, UBE2L6, CDC16, CBL, UBE2L3, FBXW8, UBE4A, SMURF2, UBE2NL, UBA7, CUL5, HERC3, UBOX5, MGRN1, HERC2, CDC27, NEDD4, RHOBTB2, SMURF1, BRCA1, BIRC2, CDC34, CBLB, KEAP1, PIAS4, NEDD4L, DDB2, MAP3K1, DDB1, HUWE1, SIAH1, UBE2D3, UBE2G2, XIAP, TRAF6, CDC26, ANAPC5, CUL7, UBE2F, UBR5, FBXW7, UBE2O, CUL3, SYVN1, UBE2D1, CDC20, CUL4B, PRPF19, UBE2C, CUL4A, UBE2M, FBXO2, CBLC, UBA1, MDM2, UBE2U, SOCS3, UBE2S, FZR1, BIRC3, SOCS1, KLHL13, UBE2A, UBE2G1, ANAPC11, MID1
**KEGG_ASTHMA**	28	-1.857	0.0000	IL5, IL9, IL3, CCL11, FCER1A, IL10, **MS4A2, CD40LG, HLA-DRB5, HLA-DOA, HLA-DPB1, EPX, HLA-DPA1, HLA-DRB1, PRG2, TNF, HLA-DQA2, RNASE3, HLA-DOB, IL13, HLA-DMB, IL4, FCER1G, HLA-DQA1, HLA-DRA, HLA-DQB1, CD40, HLA-DMA**
**KEGG_AUTOIMMUNE_THYROID_DISEASE**	50	-1.800	0.0038	TSHR, HLA-E, HLA-C, IFNA5, IL5, IFNA13, FAS, IFNA21, HLA-A, HLA-F, IFNA16, IFNA14, HLA-B, IFNA1, IFNA4, IFNA10, CD28, CGA, **IFNA6, IL10, CD80, CD86, HLA-G, PRF1, CD40LG, IFNA17, IFNA2, HLA-DRB5, CTLA4, HLA-DOA, TPO, IFNA7, HLA-DPB1, TSHB, FASLG, HLA-DPA1, HLA-DRB1, GZMB, IL2, IFNA8, HLA-DQA2, HLA-DOB, HLA-DMB, TG, IL4, HLA-DQA1, HLA-DRA, HLA-DQB1, CD40, HLA-DMA**
**KEGG_TYPE_I_DIABETES_MELLITUS**	41	-1.866	0.0057	HLA-E, HLA-C, IL12A, GAD2, IFNG, FAS, HLA-A, HLA-F, IL1A, INS, IL12B, HLA-B, CPE, CD28, PTPRN, LTA, **CD80, ICA1, CD86, HLA-G, PRF1, HLA-DRB5, HLA-DOA, HSPD1, HLA-DPB1, FASLG, HLA-DPA1, HLA-DRB1, GAD1, GZMB, IL2, TNF, HLA-DQA2, HLA-DOB, PTPRN2, HLA-DMB, HLA-DQA1, HLA-DRA, HLA-DQB1, IL1B, HLA-DMA**
**KEGG_HEMATOPOIETIC_CELL_LINEAGE**	85	-1.846	0.0074	ITGA4, FLT3, CR2, ITGA6, IL6R, ITGA2, CD38, CD59, EPO, CD8B, GP9, CD9, CD2, CD3E, IL5, IL11RA, ITGA1, GP1BA, GP5, IL7R, CD3G, IL5RA, IL3, CD4, IL9R, CD33, IL1A, DNTT, TFRC, GYPA, ITGA3, MS4A1, CSF2, CD1B, CD55, CD8A, **IL1R2, CSF1R, IL2RA, CD7, CD34, CD3D, IL3RA, IL4R, KIT, HLA-DRB5, MME, TPO, ITGA2B, CD22, CD1E, CSF1, CSF3R, IL1R1, HLA-DRB1, CR1, CD14, ITGB3, ANPEP, CSF2RA, TNF, FLT3LG, ITGA5, CD19, KITLG, FCGR1A, IL4, IL11, GP1BB, IL7, CD36, CD5, CD1C, CD1A, ITGAM, HLA-DRA, FCER2, CSF3, IL1B, THPO, IL6, EPOR, CD1D, CD44, CD37**
**KEGG_ALLOGRAFT_REJECTION**	35	-1.813	0.0074	HLA-E, HLA-C, IL12A, IL5, IFNG, FAS, HLA-A, HLA-F, IL12B, HLA-B, CD28, **IL10, CD80, CD86, HLA-G, PRF1, CD40LG, HLA-DRB5, HLA-DOA, HLA-DPB1, FASLG, HLA-DPA1, HLA-DRB1, GZMB, IL2, TNF, HLA-DQA2, HLA-DOB, HLA-DMB, IL4, HLA-DQA1, HLA-DRA, HLA-DQB1, CD40, HLA-DMA**
**KEGG_MATURITY_ONSET_DIABETES_OF_THE_YOUNG**	25	-1.589	0.0225	NR5A2, FOXA2, HNF4A, BHLHA15, PAX4, HNF1A, NEUROD1, PDX1, SLC2A2, GCK, INS, NKX6-1, IAPP, ONECUT1, NKX2-2, **PKLR, HNF4G, PAX6, HNF1B, MNX1, NEUROG3, FOXA3, MAFA, HES1, HHEX**
**KEGG_NOD_LIKE_RECEPTOR_SIGNALING_PATHWAY**	60	-1.608	0.0236	MAPK8, MAP3K7, NOD1, NAIP, TAB2, TAB3, MAPK1, CHUK, CASP8, MAPK9, CARD18, MAPK14, CARD8, CARD6, MAPK3, TAB1, BIRC2, PYDC1, IKBKB, XIAP, TRAF6, MAPK13, SUGT1, NOD2, NFKBIB, NLRP1, HSP90AB1, CCL11, CCL8, CCL13, CCL7, **MAPK10, CCL2, CCL5, MEFV, IL18, RIPK2, CASP1, HSP90B1, PSTPIP1, MAPK12, RELA, TRIP6, BIRC3, MAPK11, TNF, NLRC4, NFKB1, TNFAIP3, CXCL1, CARD9, CXCL2, HSP90AA1, IKBKG, CASP5, NLRP3, IL1B, PYCARD, IL6, NFKBIA**
**KEGG_GRAFT_VERSUS_HOST_DISEASE**	38	-1.640	0.0342	KLRC1, HLA-E, KIR2DL1, HLA-C, KIR3DL1, IFNG, FAS, HLA-A, KIR3DL2, HLA-F, IL1A, HLA-B, KIR2DL3, CD28, **CD80, CD86, HLA-G, PRF1, HLA-DRB5, HLA-DOA, HLA-DPB1, FASLG, HLA-DPA1, HLA-DRB1, KLRD1, GZMB, IL2, TNF, HLA-DQA2, HLA-DOB, HLA-DMB, KIR2DL2, HLA-DQA1, HLA-DRA, HLA-DQB1, IL1B, IL6, HLA-DMA**

**KEGG_LEISHMANIA_INFECTION**	69	-1.686	0.0367	ITGA4, STAT1, MARCKSL1, MAP3K7, TAB2, CYBA, TGFB2, ELK1, MAPK1, TLR4, FOS, MAPK14, ITGB1, IL12A, MAPK3, TAB1, MYD88, IFNG, TGFB3, PTGS2, JAK1, IRAK4, JUN, IFNGR1, TRAF6, IL1A, MAPK13, C3, IFNGR2, IL12B, NOS2, NFKBIB, JAK2, IL10, FCGR3B, FCGR3A, HLA-DRB5, HLA-DOA, **MAPK12, RELA, HLA-DPB1, HLA-DPA1, HLA-DRB1, CR1, MAPK11, TLR2, TNF, ITGB2, HLA-DQA2, HLA-DOB, NCF1, NFKB1, PRKCB, HLA-DMB, FCGR1A, IL4, TGFB1, HLA-DQA1, ITGAM, HLA-DRA, HLA-DQB1, PTPN6, IRAK1, NCF2, FCGR2A, IL1B, NCF4, HLA-DMA, NFKBIA**
**KEGG_SYSTEMIC_LUPUS_ERYTHEMATOSUS**	131	-1.675	0.0388	HIST1H4L, TRIM21, HIST2H4A, HIST3H3, C9, SNRPD3, HIST1H3E, C5, SSB, HIST1H4E, H2AFV, H2AFY, HIST1H4H, HIST2H2BE, HIST2H3C, ACTN4, C2, C4B, SNRPD1, C4A, H2BFWT, IFNG, H2AFJ, H3F3A, HIST1H2AK, HIST1H4C, GRIN2B, HIST2H4B, HIST1H4I, HIST1H4K, C6, HIST1H4D, HIST1H2BN, C1QB, H2AFY2, HIST1H2AB, H2AFZ, HIST1H2AA, HIST2H2AB, GRIN2A, C3, HIST1H2BL, HIST2H3A, HIST4H4, HIST1H2BB, HIST1H2AD, HIST1H2BE, HIST2H2BF, CD28, H3F3C, HIST1H2BO, C1QC, C8G, HIST1H2AG, HIST1H3G, HIST1H4F, C1QA, HIST1H2BC, HIST1H2AC, HIST3H2BB, H2AFB2, HIST2H3D, C8B, HIST2H2AA3, **IL10, HIST1H3A, HIST1H2BF, HIST2H2AC, CD80, C8A, HIST1H2AI, FCGR3B, CD86, ACTN2, FCGR3A, C1S, H2AFX, CD40LG, HIST1H2BJ, HIST2H2AA4, HIST1H2BD, HIST1H4B, HIST3H2A, HIST1H2BK, SNRPB, HIST1H4J, HLA-DRB5, HIST1H2AH, HLA-DOA, HIST1H4A, HIST1H2AM, C7, C1R, H2AFB3, HIST1H3H, HLA-DPB1, H2AFB1, HIST1H3D, HIST1H4G, H2BFM, HLA-DPA1, HIST1H2AE, HIST1H2BA, HLA-DRB1, ACTN1, HIST1H2BG, HIST1H3B, TNF, HLA-DQA2, HLA-DOB, HIST1H2BI, HLA-DMB, FCGR1A, FCGR2B, HLA-DQA1, HIST1H2BM, HLA-DRA, HLA-DQB1, H3F3B, HIST1H2AJ, CTSG, HIST1H3C, ELANE, FCGR2A, HIST1H2BH, HIST1H2AL, HIST1H3J, HIST1H3I, CD40, HIST1H3F, HLA-DMA**

* Core Enrichment genes are shown in bold.

Table S7. Down-modulated (blue) and up-regulated (red) genes resulting from SAM analyses comparing ST3GAL6-AS1 silenced and scramble NCI-H929 and LP1 cell lines, ordered based on (d)-score. Genes at *q*-value = 0 were marked in bold.

Gene Name	Score(d)	Fold Change	Gene Name	Score(d)	Fold Change
**RAP1A**	**−9.361**	**0.054**	PLD4	3.638	2.945
**MKI67**	**−5.298**	**0.306**	TEC	3.628	3.644
**MYO1D**	**−4.948**	**0.217**	C1orf53	3.537	2.687
**STX8**	**−4.776**	**0.366**	LGMN	3.476	1.779
**NSUN2**	**−4.744**	**0.242**	SYT11	3.429	4.098
**STXBP1**	**−4.657**	**0.223**	MAML2	3.342	2.187
**KDM5B**	**−4.372**	**0.244**	RP11-195F19.5	3.043	4.041
**PTPRS**	**−4.289**	**0.127**	ELOVL4	3.022	1.956
**SMYD3**	**−4.187**	**0.331**	PCGF3	3.004	1.997
**CBFA2T2**	**−4.018**	**0.392**	SMPD1	2.868	2.380
**YBEY**	**−4.009**	**0.217**	ZNF554	2.858	2.184
**SERPINA1**	**−3.642**	**0.510**	CFAP46	2.853	1.935
**MTM1**	**−3.588**	**0.363**	NPIPB8	2.807	2.237
**SLC16A10**	**−3.548**	**0.182**	RAD51D	2.727	2.366
**CDKN3**	**−3.394**	**0.419**	IFNLR1	2.704	2.116
**POLR1A**	**−3.265**	**0.535**	IL17RA	2.666	2.016
SUPT6H	−3.192	0.440	CTSO	2.637	2.067
CCNH	−3.153	0.317	TOB1	2.617	2.151
CCDC14	−3.089	0.319	HAGH	2.617	2.223

SAMD12	−2.994	0.282
KLRG2	−2.972	0.528
FAM188B	−2.914	0.369
BLM	−2.911	0.524
CHCHD3	−2.909	0.454
ZDHHC14	−2.858	0.396
FTO	−2.843	0.504
RIN3	−2.826	0.449
CTC-295J13.3	−2.790	0.544
A1BG	−2.769	0.198
BCL7C	−2.710	0.481
FHIT	−2.683	0.267
CADM1	−2.681	0.437
C14orf159	−2.676	0.497
ANKMY2	−2.675	0.254
PRKDC	−2.594	0.604
USP34	−2.572	0.514
HS6ST1	−2.538	0.439
TANK	−2.446	0.301
RBM47	−2.400	0.553
OR8K1	−2.399	0.341
EPB41L3	−2.395	0.525

WHAMM	2.616	1.739
DUSP3	2.605	2.049
SMKR1	2.575	2.352
SLC25A20	2.561	1.626
SELPLG	2.535	1.801
BAMBI	2.533	2.257
PLEKHA3	2.517	2.526
TCEAL4	2.515	1.819
PCBP4	2.503	1.830
NPHP1	2.447	1.998
SIDT2	2.446	1.870
WSB2	2.435	2.003
FBXO10	2.434	2.564
BASP1	2.424	2.265
CSNK1E	2.403	1.744
C14orf79	2.388	2.134
TST	2.385	1.918
UBE2M	2.382	2.951
DCAF11	2.381	1.961
GADD45B	2.374	1.832
TMEM255A	2.361	2.405
FAM127A	2.361	3.607
SIAE	2.335	1.813
CTSA	2.320	1.998
THBS3	2.319	1.991
MAN1B1	2.300	2.097
DNASE2	2.298	1.811
VAPA	2.293	1.787
DNAAF3	2.290	3.761
CAMK2D	2.278	1.601
DCAKD	2.278	1.750
LLGL1	2.249	1.711
DDIT4	2.226	2.184
RNFT1	2.223	3.089
IL10RA	2.215	2.334
NPTN	2.202	2.006
PGPEP1	2.188	1.741
MAGED2	2.181	1.716
TSPAN2	2.181	1.684
LRRC14B	2.178	1.744

CCDC92	2.175	1.680
CSRNP2	2.170	1.758
NCOA7	2.169	1.967
LRRC41	2.164	1.654
CHPF	2.157	2.341
RFFL	2.153	1.970
PSEN2	2.151	1.670
PIGS	2.149	1.949
CCDC9	2.147	1.875
APITD1	2.142	1.902
NCSTN	2.142	1.538
UBE2QL1	2.139	1.895
ANKRD42	2.139	1.708
FUCA1	2.135	2.015
CCT6B	2.134	2.580
F11R	2.131	1.647
CCNYL1	2.129	2.023
TMEM161A	2.127	1.875
LPCAT4	2.125	2.201
ATP6AP1	2.122	1.562
ZNF558	2.116	2.122
RABGEF1	2.111	1.730
AKIRIN2	2.105	1.491
YLPM1	2.105	1.836
CLPTM1	2.104	1.916
NAP1L2	2.095	1.713
COQ6	2.095	1.649
RPAIN	2.095	1.722
SEC23A	2.091	1.826
ZSWIM8	2.084	1.693
LYSMD4	2.082	2.023

Table S8. List of the significantly enriched Annotation Cluster for the 131 differentially expressed coding genes (Enrichment Score >1.3) by DAVID 6.8 Bioinformatics tool.

Annotation Cluster	Enrichment Score	GO Term	Count	%	*p*-Value	Genes
**Annotation Cluster 1**	**1.930**	**GO:0042987~amyloid precursor protein catabolic process**	3	2.33	0.008	NCSTN, CSNK1E, PSEN2
**Annotation Cluster 2**	**1.601**	**GO:0000079~regulation of cyclin-dependent protein serine/threonine kinase activity**	4	3.10	0.023	BLM, CCNH, CCNYL1, CDKN3
**Annotation Cluster 3**	**1.562**	**GO:0017157~regulation of exocytosis**	6	4.65	0.007	ATP6AP1, SYT11, STXBP1, RABGEF1, RAP1A, LLGL1

**Annotation Cluster 4**	**1.486**	**GO:0012501~programmed cell death**	20	15.50	0.040	FHIT, CADM1, LGMN, TMEM161A, STXBP1, PRKDC, RFFL, DDIT4, NCSTN, DNASE2, EPB41L3, PCBP4, CSRNP2, UBE2M, PSEN2, SMPD1, CAMK2D, GADD45B, FBXO10, BCL7C
**Annotation Cluster 5**	**1.468**	**GO:0010648~negative regulation of cell communication**	16	12.40	0.013	LGMN, SYT11, TMEM161A, STXBP1, RFFL, CBFA2T2, TANK, DDIT4, MTM1, DUSP3, CSNK1E, SMPD1, RABGEF1, RAP1A, BAMBI, TOB1
**Annotation Cluster 6**	**1.409**	**GO:0035335~peptidyl-tyrosine dephosphorylation**	4	3.10	0.029	MTM1, DUSP3, PTPRS, CDKN3
**Annotation Cluster 7**	**1.304**	**GO:0045936~negative regulation of phosphate metabolic process**	9	6.98	0.030	DUSP3, CSRNP2, MYO1D, SMPD1, SMYD3, RABGEF1, PRKDC, GADD45B, DDIT4

Table S9. C6 and KEGG gene sets obtained using Gene Sets Enrichment Analysis showing significant modulation between ST3GAL6-AS1 KD HMCLs and control. (NES >1.5 or <−1.5; p-value <0.05, NES: Normalized Enrichment Score).

NAME	SIZE	NES	NOM *p*-val	Gene list *
KEGG_LYSOSOME	121	2.08	0.0000	**CD68, NAGLU, ARSA, CTSF, HEXA, ABCA2, SMPD1, CTSS, FUCA1, LAPTM5, AP4S1, TCIRG1, MAN2B1, CTSD, CTSO, GAA, CTSH, AP3S2, ACP2, PLA2G15, SORT1, GNPTG, IDUA, CTSB, DNASE2, CTSZ, AP4M1, SLC11A1, CTSA, SGSH, GALC, CTSL, IDS, ATP6V0B, LGMN, CTSK, ATP6V0D1, AP4B1, LAMP2, HEXB, MCOLN1, HGSNAT, NAPSA, ABCB9, ARSG, PPT2, NEU1, NAGA, M6PR, CTSV, CTNS, AP3B2, ATP6AP1, ATP6V0A2, AP1G1, SUMF1, LAMP1, AP3D1, AP1B1, ATP6V1H, GNPTAB, AP1M1, PSAP, ATP6V0C, NAGPA, NPC1, CLTA, CD63, SLC17A5, AP1S1, ATP6V0A1, ARSB, NPC2, CLTB**, GGA3, GLA, GUSB, GGA1, GBA, CLN5, AP3M2, IGF2R, AP1S3, LAPTM4B, SLC11A2, GNS, ENTPD4, GALNS, GGA2, AP3B1, SCARB2, TPP1, AGA, CTSG, ASAH1, CD164, PPT1, HYAL1, LAPTM4A, CLTC, GLB1, CTSC, MANBA, CLTCL1, AP4E1, LAMP3, MFSD8, AP3M1, CTSE, AP1M2, CTSW, GM2A, DNASE2B, ACP5, PSAPL1, CLN3, AP3S1, ATP6V0D2, LIPA, AP1S2, ATP6V0A4
KEGG_GLYCOSYLPHOSPHATIDYLINOSITOL_GPI_ANCHOR_BIOSYNTHESIS	24	1.75	0.0000	**GPAA1, PIGQ, PIGZ, PIGS, PIGV, PIGC, GPLD1, PIGO, PIGW, PIGN, DPM2, PIGT, PIGU, PIGL**, PIGM, PGAP1, PIGP, PIGB, PIGF, PIGX, PIGK, PIGA, PIGH, PIGG
KEGG_NOTCH_SIGNALING_PATHWAY	46	1.705	0.0015	**MAML2, JAG1, DTX3, DTX3L, PSEN2, DLL1, MFNG, HDAC2, DTX2, NOTCH2, NOTCH4, NUMBL, NOTCH1, KAT2A, NCSTN, MAML1, DLL3, PSEN1, CTBP1, EP300**, DVL2, PSENEN, CIR1, CREBBP, DVL3, ADAM17, NUMB, NCOR2, DLL4, PTCRA, HDAC1, RBPJL, JAG2, DTX4, HES1, DTX1, NOTCH3, RBPJ, SNW1, DVL1, MAML3, LFNG, HES5, KAT2B, RFNG, APH1A
KEGG_CITRATE_CYCLE_TCA_CYCLE	29	1.665	0.0047	**SDHD, PCK1, DLST, OGDHL, PC, ACO2, MDH2, SUCLA2, OGDH**, ACO1, SUCLG1, MDH1, IDH3B, CS, PCK2, IDH1, SUCLG2, PDHB, ACLY, IDH2, IDH3G, SDHC, IDH3A, FH, DLAT, DLD, PDHA1, PDHA2, SDHB
KEGG_N_GLYCAN_BIOSYNTHESIS	46	1.62	0.0105	**ALG9, MAN1B1, RPN1, DOLPP1, ALG10B, B4GALT1, ALG12, MAN1C1, B4GALT2, MGAT2, ALG1, MGAT4B, MGAT4A, DPM2, ST6GAL1, ALG3, MGAT1, ALG10, MOGS, ALG13, B4GALT3, MAN2A2, DPM3**, ALG6, GANAB, DPAGT1, RFT1, DDOST, MAN2A1, MGAT5B, ALG5, MAN1A1, DAD1, MAN1A2, FUT8, ALG11, STT3A, ALG8, RPN2, ALG2, DPM1, STT3B, ALG14, MGAT5, TUSC3, MGAT3
KEGG_UBIQUITIN_MEDIATED_PROTEOLYSIS	128	1.59	0.0026	**UBE2M, UBOX5, UBE2W, UBE2N, UBE2E3, CDC34, UBA7, UBE2QL1, PIAS3, WWP2, CUL7, SYVN1, PML, DDB2, TRIM32, FBXO4, UBE2A, ANAPC2, KEAP1, UBE2O, UBE2D4, PIAS4, MID1, UBE2E2, HERC2, UBE2L3, UBE2C, UBE2J2, FBXW8, PPIL2, ANAPC7, SMURF2, BIRC2, RNF7, ANAPC11, WWP1, FZR1, RBX1, HERC4, CBL, ANAPC13, UBA1, STUB1, UBE2L6, RCHY1, SAE1, UBE3B, RHOBTB2, CDC16**, KLHL9, MGRN1, HERC3, UBA2, DET1, HERC1, UBE2D3, ANAPC4, UBE2H, CDC20, MAP3K1, NEDD4, UBE2Z, UBE2U, UBE3C, UBE2E1, TRAF6, CUL4A, SOCS3, DDB1, UBE2K, UBR5, UBE2Q2, UBE3A, ERCC8, FBXO2, CDC23, FBXW11, UBA3,

				SIAH1, KLHL13, CDC26, UBE2G1, ANAPC5, UBE2Q1, UBE2R2, VHL, NHLRC1, UBE2F, PRPF19, ANAPC10, TRIP12, HUWE1, CUL2, UBE2G2, SOCS1, CDC27, ITCH, FANCL, UBE2J1, BTRC, UBE2D2, CBLB, TRIM37, CUL1, UBE4B, UBE4A, ANAPC1, CUL4B, AIRE, SKP2, PIAS2, CUL3, UBE2D1, MDM2, CUL5, BIRC6, PIAS1, UBE2I, FBXW7, UBA6, UBE2B, NEDD4L, BRCA1, CBLC, SKP1, BIRC3, XIAP, SMURF1
KEGG_OTHER_GLYCAN_DEGRADATION	16	1.59	0.0149	**HEXA, NEU3, FUCA1, MAN2B1, MAN2C1, MAN2B2, HEXB, NEU1**, ENGASE, FUCA2, GBA, AGA, GLB1, MANBA, NEU4, NEU2
KEGG_PANTOTHENATE_AND_COA_BIOSYNTHESIS	16	1.57	0.0243	**PANK1, ENPP3, BCAT1, PPCDC, COASY, PPCS**, VNN2, PANK2, BCAT2, PANK4, PANK3, VNN1, ENPP1, UPB1, DPYD, DPYS
KEGG_PEROXISOME	78	1.57	0.0099	**SLC27A2, ABCD1, AMACR, XDH, ACAA1, MLYCD, PXMP4, PHYH, PEX14, ACSL1, HMGCL, PEX12, ACOT8, GSTK1, PEX7, PEX2, PEX16, CRAT, ABCD2, ACOX1, EHHADH, ACOX3, ABCD4, PEX11A, PEX19, PEX11B, PECR, ABCD3, MVK, PXMP2, HACL1, PEX26, EPHX2, MPV17, ECH1, PRDX1, PRDX5, PEX5, CROT, PMVK, PEX1, DECR2, PAOX**, PEX13, FAR2, ECI2, PEX6, SCP2, SOD2, SLC25A17, NUDT19, IDH1, NOS2, PEX3, SOD1, IDH2, CAT, ACSL5, PEX10, DDO, BAAT, PEX11G, DHRS4, AGPS, ACSL4, HSD17B4, GNPAT, NUDT12, ACSL3, PIPOX, HAO2, FAR1, DAO, HAO1, AGXT, ACOX2, MPV17L, ACSL6
KEGG_VALINE_LEUCINE_AND_ISOLEUCINE_DEGRADATION	43	1.57	0.0119	**BCKDHA, ALDH6A1, BCAT1, ACAD8, ACAA1, IVD, HMGCL, AOX1, ABAT, HIBADH**, EHHADH, DBT, ALDH1B1, MCEE, ECHS1, HADH, MCCC1, PCCA, BCAT2, ACADS, HMGCS1, AUH, ACAT2, ALDH9A1, PCCB, ACAA2, HADHB, HSD17B10, OXCT2, HADHA, ACAT1, DLD, ALDH3A2, MCCC2, OXCT1, ACADM, ALDH7A1, ACADSB, BCKDHB, HMGCS2, HIBCH, IL4I1, ALDH2
KEGG_INOSITOL_PHOSPHATE_METABOLISM	53	1.53	0.0188	**PIKFYVE, ALDH6A1, INPP5E, INPP4B, PIP4K2C, ITPKB, INPP5A, PLCG2, INPPL1, ISYNA1, PIK3C2G, PIK3CD, PIP5K1C, CDIPT, PIK3C2B, OCRL, SYNJ1, PI4KB, IPPK, PIK3CB, PIK3CG, PTEN, INPP5K, PIP4K2B, PLCD3, INPP1, PIP4K2A, MINPP1, PI4KA**, INPP4A, MIOX, IPMK, PIK3C2A, PLCB3, PLCD4, ITPK1, PLCE1, PIK3C3, INPP5J, IMPA1, PIK3CA, PIP5K1B, ITPKA, PLCB4, IMPA2, PLCB2, INPP5B, TPI1, SYNJ2, PLCG1, PLCD1, PLCZ1, PLCB1
KEGG_GLYCEROPHOSPHOLIPID_METABOLISM	75	1.52	0.0104	**MBOAT7, CHKB, LPCAT4, ACHE, PLPP3, CHPT1, PLA2G15, PGS1, ETNK1, PEMT, PHOSPHO1, TAZ, PTDSS2, LPCAT1, DGKQ, PLD2, PLPP2, PCYT2, AGPAT1, CDIPT, CDS2, LPCAT3, ADPRM, LCAT, MBOAT2, GPAT3** PISD, DGKA, CHKA, DGKG, GPAT4, LPCAT2, DGKE, AGPAT2, PLA2G4A, CRLS1, GPAM, GPD2, GPAT2, CDS1, PCYT1A, AGPAT3, GPD1L, PLA2G12A, DGKD, MBOAT1, LYPLA2, PLA2G2E, LYPLA1, DGKI, PLA2G2F, LPGAT1, PLA2G6, PLA2G1B, PTDSS1, DGKB, PLA2G3, GNPAT, LCLAT1, PLA2G12B, CHAT, PLA2G10, AGPAT4, PLA2G2C, DGKZ, DGKH, PLA2G4E, PLPP1, ETNK2, PCYT1B, PLD1, GPD1, PLA2G2D, PLA2G5, PLA2G2A
KEGG_SPHINGOLIPID_METABOLISM	38	1.51	0.0241	**ARSA, NEU3, SMPD1, PLPP3, SGPP1, SPTLC2, SGPL1, GALC, ACER3, UGT8, PLPP2, NEU1, SPHK2, B4GALT6, DEGS1, SMPD3, ACER2, ASAH2** SMPD2, GLA, ACER1, GBA, SPHK1, KDSR, SGMS2, UGCG, SPTLC1, ASAH1, GLB1, ENPP7, CERK, SGMS1, SGPP2, PLPP1, NEU4, NEU2, DEGS2, GAL3ST1
KEGG_PHENYLALANINE_METABOLISM	17	−2.16	0.0000	MIF, ALDH3B2, GOT1, AOC2, ALDH1A3, PRDX6, GOT2, AOC3, DDC, **MAOA, HPD, IL4I1, ALDH3B1, ALDH3A1, MAOB, PAH, TAT**
KEGG_OLFACTORY_TRANSDUCTION	345	−1.98	0.0000	OR2K2, OR1N2, OR4M1, OR4C46, OR2A1, CALM1, OR8G1, OR2T4, OR2T12, GUCA1C, OR2A4, OR6B2, OR10H4, OR1L3, OR2B6, OR10P1, OR4K17, OR2L3, OR5B17, PRKACB, OR2G6, CAMK2D, OR1L8, OR8I2, OR1Q1, OR1C1, OR6B1, OR51T1, OR5J2, OR2M7, OR7G2, OR2W3, OR51A4, OR10H5, OR4K14, OR14C36, OR4L1, OR1E2, OR51Q1, OR13A1, OR8B12, OR2F2, OR52D1, OR13F1, OR5B21, OR1G1, OR2AK2, OR6C65, OR52E2, OR9G1, OR10Q1, GUCY2D, OR8U1, OR51B4, OR7D2, OR8H3, GRK3, OR5AK2, OR14J1, OR4C12, OR6N2, OR10J1, OR1A1, OR2M2, OR52E4, OR6C68, ADCY3, OR4E2, OR6C76, OR51E1, OR13C4, OR5M8, OR6C6, PRKACA, OR52I1, OR4D6, OR52A1, OR10G7, OR2S2, OR13J1, OR4F5, OR4D1, GUCA1A, OR1J2, OR8B4, OR5P2, OR10G9, OR2T8, OR5C1, OR51I1, OR14I1, OR4K1, OR2W1, OR6F1, OR52B2, OR6K3, OR5H6, OR10H3, OR4D9, OR4K2, OR9I1, OR5P3, CALM3, OR5A1, OR8D4, OR6V1, OR1K1, OR51A7, OR8B8, OR5AP2, GUCA1B, OR11H1, CALML6, OR7G1, OR1J4, OR51F1, OR13C8, OR2H1, OR4C11, PDE1C, OR4S2, OR10V1, OR52B6, OR10G2, OR51F2, OR5K1, OR1L1, OR6C2, OR2T6, OR8J3, OR1E1, CLCA4, OR8K5, OR2T1, OR5F1, OR5V1, PRKG2, OR51L1, OR6N1, OR13C3,

				OR12D3, OR51D1, OR52M1, OR5D16, OR2AG1, OR4B1, OR4C3, OR52W1, PRKACG, OR5M1, CAMK2B, OR5B3, OR1S2, OR5T1, OR4N5, OR6M1, OR10A3, OR2J3, OR4A15, OR9G4, OR10A4, OR6C1, OR4D10, OR2Y1, OR5AN1, OR10T2, CNGA4, OR3A1, OR10Z1, OR14A16, OR1D2, OR6Q1, OR4K13, OR52N2, OR4D5, OR3A3, OR6C3, OR6C4, OR1F1, OR11L1, OR9K2, OR10K1, OR2AG2, OR2V2, OR52J3, OR7A5, OR13H1, OR51G2, OR5A2, OR7E24, OR6Y1, CALM2, OR10AD1, OR10R2, OR56A3, OR2A12, OR6B3, OR51M1, OR4C15, OR7A10, OR8S1, OR1M1, ARRB2, OR52K1, OR4A47, OR4K5, GNAL, OR2H2, OR4F6, OR10J3, OR9Q1, OR51A2, OR5H2, OR10J5, OR10G3, OR2M4, OR52I2, OR2T29, OR8G5, OR52A5, **OR2D2, OR11H6, OR6C74, OR9Q2, OR10A7, CLCA1, OR2C3, OR11G2, OR4D11, OR2T27, OR2D3, OR10AG1, OR5AU1, OR5M11, OR52N1, OR13C9, OR2A2, OR51E2, OR4C13, OR2T3, OR13G1, OR6C70, OR2T5, OR4A16, OR2C1, OR6S1, OR6K2, OR9A4, OR5AC2, OR52E8, OR5L2, OR4P4, OR1D5, OR10K2, CAMK2G, OR56A4, OR4X1, CLCA2, OR10C1, OR2L13, OR11H4, OR51B6, OR2B2, OR1I1, OR9A2, OR5AS1, OR51I2, OR2AT4, OR2G2, OR5D18, OR5D14, OR1J1, OR1A2, OR5K2, CALML5, OR2A42, OR2B3, OR8H2, CAMK2A, OR2T10, OR52H1, OR10A2, OR56A1, OR6C75, OR5M9, OR51V1, OR1L6, OR10G4, OR5I1, CALML3, CNGA3, OR2AE1, OR6K6, OR6T1, OR4D2, OR8H1, OR8A1, OR4A5, OR13D1, OR2Z1, OR4F15, OR10A5, OR1N1, OR5B12, OR4N2, OR2A5, OR56B1, OR2G3, OR52L1, OR5M3, OR10S1, OR2J2, OR6X1, OR52E6, OR1L4, OR5T2, OR2A14, OR7D4, OR56A5, OR52N4, OR2A25, OR8D1, OR7C2, OR2M5, OR4K15, OR51S1, OR11A1, OR2B11, OR10H2, OR7A17, OR7G3, OR4S1, OR52K2, OR6A2, OR5T3, OR52N5, OR56B4, OR8J1, OR10G8, PDC, OR5M10, PRKG1, OR4C6, CNGB1, OR8K1**
KEGG_COMPLEMENT_AND_COAGULATION_CASCADES	67	−1.90	0.0000	PROS1, CR1, C2, MASP1, CFI, F13A1, F12, CD59, C8G, CFD, CFH, CR2, C1QB, C1R, F8, F2R, F7, C6, SERPIND1, CD46, C4BPA, F3, SERPINE1, F2, F5, FGG, C1QC, BDKRB1, C4BPB, CD55, F11, PLAU, PLAT, PLAUR, C1QA, CPB2, **C1S, C3, MBL2, C9, KNG1, C8B, SERPINC1, KLKB1, C8A, SERPING1, PROC, F10, SERPINF2, F13B, THBD, TFPI, MASP2, PLG, C7, FGB, VWF, C5, SERPINA5, F9, FGA, CFB, C3AR1, BDKRB2, C5AR1, A2M, SERPINA1**
KEGG_ARACHIDONIC_ACID_METABOLISM	54	−1.84	0.0000	PTGS2, ALOX5, ALOX12B, PTGES2, CYP2U1, EPHX2, GPX4, AKR1C3, CBR1, PLA2G4A, GPX7, CBR3, PTGIS, CYP2B6, PLA2G12A, GGT1, GPX6, PLA2G2E, LTA4H, PLA2G2F, PLA2G6, PLA2G1B, GGT7, GGT5, GPX2, GGT6, PTGDS, CYP2J2, PLA2G3, GPX1, PTGS1, PLA2G12B, ALOX12, PLA2G10, ALOX15B, PLA2G2C, CYP2C8, CYP2C18, PLA2G4E, **CYP4A11, GPX3, CYP2E1, PTGES, TBXAS1, GPX5, CYP4F3, ALOX15, CYP4F2, CYP2C19, PLA2G2D, CYP4A22, PLA2G5, PLA2G2A, HPGDS**
KEGG_LEUKOCYTE_TRANSENDOTHELIAL_MIGRATION	113	−1.56	0.0000	TXK, ITK, CTNND1, VAV2, F11R, RAC2, JAM3, ITGAM, CLDN6, PLCG2, ESAM, PIK3CD, MYL12A, PIK3R1, ACTB, CDC42, CLDN15, ITGAL, MAPK13, JAM2, VAV1, PIK3CB, SIPA1, PIK3CG, PXN, CTNNA2, CLDN14, MAPK12, CYBA, ACTN1, CLDN4, RHOH, RAPGEF3, GNAI2, NCF4, CLDN7, CLDN16, BCAR1, CLDN11, VCL, CTNNB1, ACTN4, PTK2B, PRKCA, CLDN20, CLDN10, CLDN3, PRKCB, CTNNA1, ACTG1, ITGB1, RASSF5, ITGA4, RAP1B, CDH5, EZR, MAPK14, PRKCG, ROCK2, CXCR4, CLDN1, ICAM1, RHOA, MMP2, PTPN11, ACTN3, ARHGAP5, MYLPF, PIK3CA, MYL12B, MSN, CLDN18, MMP9, CLDN9, CYBB, GNAI1, MYL7, NOX3, ROCK1, VASP, MYL5, RAPGEF4, CLDN5, THY1, **ARHGAP35, MYL9, ACTN2, PECAM1, PLCG1, MYL2, CLDN19, CXCL12, OCLN, PIK3R5, CLDN22, PIK3R3, CLDN2, CTNNA3, CLDN17, CLDN23, CLDN8, PIK3R2, RAC1, GNAI3, VAV3, PTK2, MAPK11, NCF2, MYL10, VCAM1, ITGB2, NOX1, RAP1A**
KEGG_LINOLEIC_ACID_METABOLISM	25	−1.93	0.0027	CYP3A43, PLA2G4A, PLA2G12A, PLA2G2E, PLA2G2F, PLA2G6, PLA2G1B, CYP2J2, PLA2G3, PLA2G12B, PLA2G10, **PLA2G2C, CYP2C8, CYP2C18, PLA2G4E, CYP1A2, CYP3A5, AKR1B10, CYP2E1, CYP3A4, ALOX15, CYP2C19, PLA2G2D, PLA2G5, PLA2G2A**
KEGG_HEMATOPOIETIC_CELL_LINEAGE	79	−1.63	0.0033	CR1, IL1B, ITGA3, THPO, ITGAM, CD3E, IL4R, CD1E, IL7, CD59, CD5, IL11RA, CD3D, IL1A, CR2, HLA-DRA, IL6, EPOR, MME, GP1BA, CD37, HLA-DRB5, IL5RA, IL1R2, CD8A, CD33, ITGA4, GP5, TFRC, CSF3, TPO, IL11, GP9, ITGA2, IL6R, ANPEP, CD38, ITGA6, IL4, ITGA2B, TNF, IL5, CSF1R, CD55, EPO, CD4, CD19, **CD9, CD2, CD44, GYPA, CD14, CD34, DNTT, FLT3, CD36, IL3, CD1A, HLA-DRB1, CD3G, CD7, CD1C, CD1D, FCER2, IL2RA, MS4A1, CSF2, KIT, ITGA5, ITGA1, IL7R, IL1R1, CD1B, CSF3R, CD8B, CSF1, KITLG, ITGB3, CD22**
KEGG_TYROSINE_METABOLISM	40	−1.61	0.0154	HGD, GSTZ1, AOX1, METTL6, PNMT, FAH, ADH6, MIF, ADH5, TH, LCMT1, ALDH3B2, HEMK1, ADH4, GOT1, AOC2, ALDH1A3, LCMT2, ADH1C, METTL2B, TPO, TRMT11, COMT, GOT2, ADH1A, AOC3, DBH, DDC, ADH7, **MAOA, ADH1B, HPD, IL4I1, ALDH3B1, ALDH3A1, TYR, MAOB, TYRP1, DCT, TAT**

KRAS.KIDNEY_UP.V1_UP	138	−1.75	0	**ARPP21, CDR1, AK5, A2M, EPB41L3, NME5, PLCB1, PCDH9, DNM3, NEBL, WIF1, PCP4, TMEM100, LIMCH1, ANK3, TRIM9, GABRB1, PEG3, CRTAM, ERC2, CHGA, CHGB, GRIA2, CALB1, KIT, CRYM, GPM6A, SLC12A5, SORBS1, RTN1, RIT2, TCEAL2, MPZL2, GRM5, PECAM1, GHRH, NSG1, MAP7, AQP4, GPNMB, NRXN1, VIPR2, TUBB4A, SLC1A3, TRIM2, SFRP1, GCGR, TRIL, PMP2**, LMO3, HEY1, RAPGEF4, NEFL, PNMA2, GABRR2, SLC6A1, NOL4, MAFB, SCN1B, SPARCL1, FGF9, CCND2, SIGLEC15, ST3GAL6, SYT1, GLDC, GABRA1, EMP1, SLC12A3, SCG3, TMOD1, NMNAT2, PLP1, KCNA5, NUDT11, CFAP69, TSPAN7, SNAP25, CRYAB, GAP43, DUSP6, SMR3A, NRN1, SV2B, ITGBL1, SLC6A15, GAD1, ZNF536, NDN, CA2, RNLS, DLGAP2, MEF2C, DDX6, CPEB3, PLSCR4, OCLM, CPE, SCG5, GATM, ANK2, FZD3, GPR19, GABRA5, SNAP91, GRIK2, HPCAL4, XK, ESRRG, NRCAM, RELN, SOBP, PLCL1, MAGEH1, GPRC5B, CAB39L, SERPINI1, ALDH1A1, GPR37, ADCY1, KCNJ8, PELI2, METTL7A, MMP27, WSCD1, ASPA, RCAN2, SORL1, DYNC1I1, ETV1, ATP1A2, CP, NAP1L2, SCN2A, SATB1, KIF5C, CDK5R1, TRIB2
KRAS.LUNG.BREAST_UP.V1_DN	134	−1.70	0	**SERPINB13, CALCB, DTNB, IVL, CRABP2, CCDC121, SCN7A, CXCL14, ANKRD1, IGFBP2, TBR1, TRIM9, GABRA4, PVALB, HRK, CYP21A2, NTF3, KRT13, VAV3, RARRES2, CLDN8, ADAMTS20, TSHB, IFNA16, ART1, CALML3, SERPINA10, HTR1E, CD6, DPT, AKAP6, NOX5, GLYAT, CALML5, PROC, THNSL2, APOB, MXRA8, CLCA2, MYL9, AKR1B10, GADD45G, S100A7, SPAG11A, SCARF1, C5AR2, DEFB1, OLFML2A, GLS2, CD36, KCNMB1, PDE3A, KRT4, PDE6B, KRT38, FLT1**, NCR3, ADGRE1, HTR1B, TM4SF4, MMP28, PCDHB1, RUNDC3B, CDH12, BDKRB1, RAG1, KRT16, VAX2, RTP4, VPS50, AMBN, CDH16, SLC12A3, COL5A2, FGFR3, FRAS1, ERAP2, CACNA1I, CD248, ADD2, HHLA1, SOX11, ALDH5A1, COBL, COL2A1, GDNF, RBM17, TRPM2, RAMP3, NOS1, SYNDIG1, TGM1, YPEL1, TAS2R4, IL5RA, UPK3B, SLC6A9, PBX1, PARP3, TRIM48, PTPRU, ACKR3, EPB41L4A, HCN2, EPHX2, SOBP, MXD3, ATP6V1B1, SPTBN2, MAST3, LGALS7, LY6D, GAMT, SLC3A1, PIK3C2B, NYNRIN, TTLL1, METTL7A, TNNI3, MAP2K6, NSUN6, KIF25, MTHFR, ALOX12B, PLBD1, DTX2, PAQR6, SIDT1, P2RX1, TNFSF10, MEGF8, IFI44L, INPP5D, RYR1
IL21_UP.V1_DN	173	−1.63	0	**ZKSCAN7, CUX2, PACRG, A1CF, PRKG1, PRB3, PCDH9, PCP4, SCN7A, IFNG, LRCH1, TINAGL1, TREM1, LAD1, NTF3, HTR3B, CEL, CIDEA, BEST2, CHGB, CCL16, A4GNT, CCK, LCN2, HKDC1, HOXB8, FGB, KCNS1, LMOD1, THEG, ZNF286A, CD177, CRYGB, GJB3, DNAH7, F13B, FRMD4A, CMKLR1, FGF16, RIN2, GPC3, POU1F1, TNP1, MAB21L1, CACNA2D3, ZNF324B, DMRT1, NRXN1, TAAR2, CFAP74, PLPPR4, SEC14L2, PDE3A, CYP3A5, PDE6B, ADTRP, LDOC1, MEOX2, GPR20, SP4, SLC17A1, NEFL, DOC2A, USP27X, MTNR1A**, ADAM7, BICDL1, ADRA1B, F11, PROX1, WNT4, NDP, ADGRL4, SLC25A31, PLCB4, MYLK, OR1F1, PHKG1, CNTNAP3B, ITGA6, AIRE, PDHA2, LATS1, ITGA2, PNMA3, TG, ZNF446, SOX3, GPX2, NACAD, CD72, TAS2R9, FAM124B, EPS8, TSPAN7, PRKG2, CD40LG, TIMP3, MISP, PDE1C, HAL, PRR5L, GUCA1B, GDNF, PPP1R3A, RAMP3, ZNF155, KDR, GCNT4, DIRAS2, IL12A, CLDN10, SLC16A2, CEP41, PLEK, GPC4, ADGB, KCNC4, SLC17A4, SLC8B1, GSR, SLC22A7, BTBD3, RALGPS2, TAS2R1, OR1A1, NPY5R, ZNF550, USP18, VWA7, KRT85, BCL2L10, KRT32, SLC17A5, LRP2BP, TFAP2B, OR2F2, LPA, ECHDC3, MPDZ, ADAM28, USP9Y, HYAL3, CORO2A, AP3B2, SPTBN4, PCDHB3, EFNA5, TNNI3, CCL24, ASPHD1, LRP3, DGKQ, IL19, FES, ACVR2A, ZNF629, RHOD, PARM1, TESMIN, OSGIN1, CHFR, CNTN2, FGF6, HBD, LRP5L, TLR5, ZNF358, CYP46A1, ACHE, PRSS16, CLEC7A, KLHDC8A
P53_DN.V2_UP	144	−1.56	0	**FZD9, TNNI3K, KRT14, PDC, CXCL6, ANGPTL3, DPYS, FGF22, MOG, AMELX, GPA33, TNR, CD69, CYP2D6, FPR3, CACNG2, FOSL2, ANXA10, MAGEC2, SPINK1, P2RY11, TNFRSF9, HAO1, HTR1E, NTSR1, IL1RAPL1, FOXL2, MMP17, CXCL12, SPRR1B, WNT3, CXCL8, SARDH, DPF1, PTX3, GSC2, PRTN3, PRKCE, SLC38A3, CLEC1A, CEACAM5, BPI, HS3ST2, MPZ, KCNMB1, CD83, ATP12A**, CYP2C8, TUBB1, IL36A, NCKAP1L, RFPL2, LGSN, ANGPTL4, CA3, EVX1, PYY, CXCL1, TNFAIP3, CLCF1, AVPR2, MT1G, CYP2A13, NFIX, SLC16A4, RGR, CDH4, LRRC32, SLC4A10, SDC4, CXCL5, RELB, INSL4, CTLA4, RND2, PKD2L1, PCDH1, C4BPA, CYP17A1, RORC, NF1, GP5, SLA, ETV5, LALBA, OSGIN2, ITM2A, RIPK4, NFKBIA, RNF39, IL1RN, NTRK3, CH25H, KRT31, OR10H3, CNOT9, PRRG3, PTK2B, POU2AF1, SYN1, RFX2, APOL3, KCNK3, INSL3, GALK1, IKZF1, PF4V1, CHRNA10, CCL4, MMP3, CHST8, CCL20, MMP13, TFAP4, BFSP2, FCRL2, GUCY2D, HIST1H1T, PRDM11, BAX, IER3, CD5L, RHD, P2RY6, PPEF1, TDRKH, EVI2A, XAF1, TP53, ME3, EML3, DLX6, PIDD1, SLC15A2, ALOX5AP, OSGIN1, TPTE, ADAM12, BARX2, PCDHA6, CXCL2, H6PD, INPP5D, CD68

KRAS.300_UP.V1_UP	139	−1.54	0	**CDR1, RNASE1, A2M, NME5, CFHR2, DNM3, ITGB2, PCP4, NTM, LRCH1, PDE2A, ASTN1, ANK3, NAV3, TOX3, MKRN3, MMP11, PEG3, CEACAM3, PRG3, CEL, EFCAB6, DNAJA4, STC1, ERC2, CHGA, CHGB, CALB1, APOD, CLUL1, HPN, FNDC8, TFPI**, RTN1, MMP1, PTX3, CD1A, CGA, FABP3, HTR7, LMO3, RBP4, HEY1, CRISP1, COL26A1, ZNF528, NOL4, SCN1B, ZP4, FGF9, SIGLEC15, ANO1, ANGPTL4, DBH, ST3GAL6, SYT1, GLDC, SLC25A31, GABRA1, KCNK7, KCNK10, SCG3, SMPX, ANPEP, PCDH8, SPP1, ITGA2, WNT7A, RDH5, PPBP, ASIC4, CXCL5, GPR4, CFAP69, G0S2, SNAP25, TAPT1, ADAM8, CSF3, DUSP6, ETV5, ARHGAP24, ITGBL1, SLC6A15, KIAA1549L, ADAM19, IL13RA2, DHRS9, RETN, RRAGD, DDX6, USH1C, TERT, GLRX, HAS2, SCG5, HIST1H2BO, GPR19, DEFB4A, SNAP91, CTNNA2, DOCK4, APOBEC3G, GABRA3, NRCAM, RELN, GBA3, MAP4K1, CXCL3, HSD11B1, ATXN3L, ETV4, BEX1, DCBLD2, EVI2A, SPRY2, IL1RL1, CEACAM1, RCAN2, SORL1, FAM155B, DYNC1I1, OR2B6, GRIN2A, KIF5A, ETV1, SLCO5A1, MCF2, ATP1A2, TFDP3, KCNH2, TRIM36, MAPK10, NAP1L2, SCN2A, KIF5C, SEMA3A, CDK5R1, TRIB2
KRAS.600_UP.V1_DN	263	−1.54	0	**CNGB1, SERPINB13, GP2, CALCB, CT55, KRT1, PYHIN1, DPYS, KRT17, DTNB, IVL, CRABP2, SCN7A, GPR52, CYP2C19, CXCL14, ANKRD1, DLK2, UGT2A3, KCNE1, SLAMF1, NAALADL1, NTF3, SCN10A, KRT13, VAV3, GKN1, ZBTB16, EDN1, HYAL4, HOXB8, LYPD3, CLDN8, SMPDL3B, ADRA2B, IFNA16, ART1, CALML3, EDAR, ACTG2, HAAO, RRAD, PCDHGB6, LRRC31, DPT, BCL11B, THBD, KLK12, CALML5, KLF3, SPRR1B, PROC, IFNA17, SERPING1, THRB, C8A, ABCG4, THNSL2, APOB, MXRA8, CLCA2, CD207, MYL9, ZNF750, RASAL1, AKR1B10, PCYT1B, GADD45G, PDE11A, CLIC3, S100A7, GPR173, PRTN3, C5AR2, COLEC10, OLFML2A, KLK7, CEACAM5, SLC5A2, TNFRSF13B, CD36, KCNMB1, NR1I3, KRT4, FGGY, PDE6B, KRT38, SDS, TREM2, ARHGEF38, CD300A, NKX6-1**, MAGIX, NCR3, MTNR1A, CYP2C18, GRM2, LFNG, MAML3, GNRH2, HTR1B, EPHB3, NOX3, AQP6, CGREF1, TM4SF4, EDN2, CKM, CHRM2, FOLR2, MUC5AC, VGLL3, ABCB1, PCDHB1, HOXC8, SIT1, YBX2, IL5, SLC22A11, IMPA2, PROP1, RUNDC3B, CTSW, CNR2, SCGB1A1, BST2, DBF4B, KRT16, RTP4, AMBN, CDH16, SCGN, KCNQ3, YOD1, EDIL3, PGLYRP1, FOXI1, RYR2, HTR1D, FGFR3, FRAS1, HSPB8, TEX15, CST4, ENPP1, DSG1, CSDC2, CACNA1I, CTAG2, CD40LG, HLA-DOA, TRH, CD248, PSORS1C1, SLC6A3, ZNF154, TUBAL3, SOX11, C1QL1, IFNA5, CCDC102B, COBL, COL2A1, SLC9A7, PODNL1, TRPM2, BBC3, RAMP3, CRYGD, KRT31, NRXN3, CIITA, C22orf31, NOS1, WNT16, TGM1, CNTFR, DSG3, TAS2R4, FGF4, CA5B, SLC29A3, APOL3, EPHB6, CLDN16, CSPG4, CATSPERB, MCHR1, TRIM48, MX1, PTPRU, GBP1, SYNPO, SLC6A14, ACKR3, MYOM2, CBL, ABCB11, MLANA, VILL, CAMK1D, NPC1L1, SPRR3, ADAMTS9, EPHX2, NR0B2, MX2, TAGLN, MXD3, ATP6V1B1, WHRN, SPTBN2, BRDT, NINJ1, TGFB2, MAST3, LGALS7, LY6D, PNMT, SLC3A1, PIK3C2B, MATN2, NYNRIN, CCR8, PIP5K1C, ZBP1, TTLL1, TNNI3, PRODH, MAP2K6, TMEM8B, XAF1, KIF25, IL19, HRH3, CHRNG, DLX6, KCNMB2, KLF8, PLBD1, APBB3, DTX2, PRRG4, DDX51, MAPRE3, ATP2A3, FCGBP, SLC30A4, CCDC33, SPATA6, UGT2B17, SIDT1, P2RX1, BLNK, PLEKHH3, TNFSF10, CADM4, ARHGAP28, DENND1C, NEU3, FETUB, PPFIA3, IFI44L
PRC2_SUZ12_UP.V1_ DN	176	−1.54	0	**SLC16A10, KALRN, CD22, SLITRK3, ST3GAL1, WFDC1, OR6A2, CACNA2D2, HOXB1, PNPLA3, CCL19, TINAGL1, CD1B, AMBP, FGA, NLGN1, HAPLN2, CACNG2, ACVRL1, PTPRO, ALDH3A1, PAX1, TM4SF20, LMOD1, HOXC5, GNGT2, ZNF695, FXYD1, UBQLN3, ADAMTS7, SERPINB4, SLC17A3, DNAI1, SOAT2, DCSTAMP, SLC12A5, APCS, PGK2, ADCY2, NPFFR1, CHRNA6, CD1D, IGLL1, FOXJ1, MYH13**, ARHGEF16, DLGAP1, PKP1, KRT3, CGA, CLEC10A, MEIS2, DRP2, ZNF696, CRISP1, ARSD, TCAP, CD247, ZWILCH, ROCK1, ACP5, RPS6KA2, KLF15, ARRB2, SHPRH, SUZ12, NPY, PF4, STAU2, ORC1, HOXA10, AP1M2, CHEK1, IL23A, LMNB2, ATP2C2, BTBD7, CCNE1, GP9, RAVER2, BCL2L14, MC5R, MAPT, DEFB126, NPY2R, PRSS8, LHX6, ZIC4, CA7, CORO1A, WRN, OPCML, TNNT2, DFFB, SRD5A2, BPNT1, HS2ST1, EVPL, BBC3, IL1RN, EPPK1, KAT8, SRSF11, C22orf31, MYF6, KLHL23, GNA15, HYAL1, DCX, RFX2, RUNDC3A, MAP2K4, PHOX2B, AGER, CBFA2T3, MAP1LC3C, POLA2, OR52A1, SLC7A6, TSPAN12, UPK1A, P2RY10, HLA-DQA1, HOXD11, FRMD8, KBTBD11, OR10J1, CLDN14, PGAP1, MARK1, RAD54L, CIB1, SLC35D1, USP2, TACR1, KLHL11, ADRB3, LDB2, EPHX2, NAA10, SLC6A6, KXD1, CXorf56, LRRC61, HAUS5, IMPG2, MED20, ELP6, ONECUT2, GLRA2, SYN3, ATG2A, NDUFC1, STXBP2, REG1A, EFNA5, MYOM1, ANK1, RECQL4, TRAC, DCC, LPCAT1, KCNMB2, CHN2, PTTG2, RITA1, RPL37A, ATP2A3, LRP5L, ATG16L1, SLC4A8, TFEC, IL10RA, CFAP70, SCHIP1, ALOXE3
CAHOY_NEURONAL	99	−1.84	0.0039	**STXBP1, KRT222, SLC17A6, NEFM, AMER3, CELF6, SYT13, PRSS12, CRHBP, CALN1, TBR1, MYO5B, SLC35F4, CCK, CALB1, MLIP, HPCA, GRP, BCL11B, NTS, GAD2, KIAA0319, COL19A1, PGM2L1, CRH, PENK**, SLC6A7, HS3ST2, FABP3, NEFL, RASGEF1A, CAMKV,

				CNTNAP4, NEUROD6, RPRML, FGF9, STX1A, SYT1, SYT5, CELF4, CACNG3, PHF24, ITPKA, HSD17B4, NXPH2, GPR22, GABRD, TRHDE, NELL1, CAMK2B, MYH3, KCNIP4, NWD2, GDA, RGS4, TSPYL5, RTN4RL1, LAMP5, DRD1, PRDM8, NAPB, CLSTN2, SV2B, HCN1, RAMP3, GREM2, CBLN4, MYT1L, NSF, JPH4, MEF2C, SATB2, LIN7B, SST, DMTN, CPNE5, EPHA7, SYT4, GABRA5, SLC6A17, ICAM5, SCG2, BCL11A, PPFIA2, ENO2, RASL10A, MAL2, METRN, GLRA2, GABRG2, SLC25A22, SLC8A1, NRGN, SH2D5, DYNC1I1, CDH9, PNOC, SIDT1, CPNE4
KRAS.50_UP.V1_UP	47	−1.63	0.0060	**CDR1, A2M, CFHR2, ITGB2, NTM, MMP11, PEG3, STC1, CHGB, CALB1, HPN, CD1A, FABP3, HTR7, LMO3, RBP4, HEY1**, FGF9, SIGLEC15, ANGPTL4, ST3GAL6, GLDC, SCG3, PCDH8, ITGA2, PPBP, CXCL5, G0S2, SNAP25, ADAM8, ETV5, KIAA1549L, IL13RA2, DHRS9, TERT, GLRX, SCG5, SNAP91, DOCK4, NRCAM, RELN, HSD11B1, IL1RL1, FAM155B, DYNC1I1, ETV1, KIF5C

* Core Enrichment genes are shown in bold.

References

1. Ronchetti, D.; Agnelli, L.; Pietrelli, A.; Todoerti, K.; Manzoni, M.; Taiana, E.; Neri, A. A compendium of long non-coding RNAs transcriptional fingerprint in multiple myeloma. *Sci. Rep.* **2018**, *8*, 6557
2. Miller, C.; Yesil, J.; Derome, M.; Donnelly, A.; Marrian, J.; McBride. K.; Auclair, D.; Keats, J.J. A comparison of clinical FISH and sequencing based FISH estimates in multiple myeloma: An MMRF CoMMpass analysis. *Blood* **2016**; *128*, 374.
3. Ritchie, M.E.; Phipson, B.; Wu, D.; Hu, Y.; Law, C.W.; Shi, W.; Smyth, G.K. limma powers differential expression analyses for RNA-sequencing and microarray studies. *Nucleic Acids Res.* **2015**
4. Subramanian, A.; Tamayo, P.; Mootha, V.K.; Mukherjee, S.; Ebert, B.L.; Gillette, M.A.; Paulovich, A.; Pomeroy, S.L.; Golub, T.R.; Lander, E.S.; et al. Gene set enrichment analysis: A knowledge-based approach for interpreting genome-wide expression profiles. *Proc. Natl. Acad. Sci. USA* **2005**, *102*, 15545–15550, doi:10.1073/pnas.0506580102.
5. Huang, D.W.; Sherman, B.T.; Lempicki, R.A. Systematic and integrative analysis of large gene lists using DAVID Bioinformatics Resources. *Nat. Protoc*. **2009**, *4*, 44–57.
6. Huang, D.W.; Sherman, B.T.; Lempicki, R.A. Bioinformatics enrichment tools: Paths toward the comprehensive functional analysis of large gene lists. *Nucleic Acids Res*. **2009**, *37*, 1–13.

© 2020 by the authors. Licensee MDPI, Basel, Switzerland. This article is an open access article distributed under the terms and conditions of the Creative Commons Attribution (CC BY) license (http://creativecommons.org/licenses/by/4.0/).
